# Extracellular vesicles derived from human ESC–MSCs target macrophage and promote anti-inflammation process, angiogenesis, and functional recovery in ACS-induced severe skeletal muscle injury

**DOI:** 10.1186/s13287-023-03530-1

**Published:** 2023-11-14

**Authors:** Xiangkang Jiang, Jingyuan Yang, Yao Lin, Fei Liu, Jiawei Tao, Wenbin Zhang, Jiefeng Xu, Mao Zhang

**Affiliations:** 1 Department of Emergency Medicine, Second Affiliated Hospital, Zhejiang University School of Medicine, No. 88 Jiefang Road, Hangzhou, 310009 Zhejiang Province China; 2 Key Laboratory of the Diagnosis and Treatment of Severe Trauma and Burn of Zhejiang Province, Hangzhou, China; 3 Zhejiang Provincial Clinical Research Center for Emergency and Critical Care Medicine, Hangzhou, China; 4 Department of Emergency Medicine, Nanjing Drum Tower Hospital, Medical School, Nanjing University, Nanjing, China

**Keywords:** Embryonic stem cells-derived mesenchymal stem cells, Extracellular vesicles, Acute compartment syndrome, Skeletal muscle injury, Macrophage, miRNA

## Abstract

**Background:**

Acute compartment syndrome (ACS) is one of the most common complications of musculoskeletal injury, leading to the necrosis and demise of skeletal muscle cells. Our previous study showed that embryonic stem cells-derived mesenchymal stem cells (ESC–MSCs) are novel therapeutics in ACS treatment. As extracellular vesicles (EVs) are rapidly gaining attention as cell-free therapeutics that have advantages over parental stem cells, the therapeutic potential and mechanisms of EVs from ESC–MSCs on ACS need to be explored.

**Method:**

In the present study, we examined the protective effects in the experimental ACS rat model and investigated the role of macrophages in mediating these effects. Next, we used transcriptome sequencing to explore the mechanisms by which ESC–MSC-EVs regulate macrophage polarization. Furthermore, miRNA sequencing was performed on ESC–MSC-EVs to identify miRNA candidates associated with macrophage polarization.

**Results:**

We found that intravenous administration of ESC–MSC-EVs, given at the time of fasciotomy, significantly promotes the anti-inflammation process, angiogenesis, and functional recovery of muscle in ACS. The beneficial effects were associated with ESC–MSC-EVs affecting macrophage polarization by delivering various miRNAs which regulate NF-κB, JAK/STAT, and PI3K/AKT pathways. Our data further illustrate that ESC–MSC-EVs mainly modulate macrophage polarization via the miR-21/PTEN, miR-320a/PTEN, miR-423/NLRP3, miR-100/mTOR, and miR-26a/TLR3 axes.

**Conclusion:**

Together, our results demonstrated the beneficial effects of ESC–MSC-EVs in ACS, wherein the miRNAs present in ESC–MSC-EVs regulate the polarization of macrophages.

**Supplementary Information:**

The online version contains supplementary material available at 10.1186/s13287-023-03530-1.

## Background

Acute compartment syndrome (ACS) is a severe musculoskeletal injury observed in clinical and characterized by a sudden and sharp increase in osseofascial pressure [1]. Timely reperfusion therapy through fasciotomy is crucial for reducing pressure-induced skeletal muscle injury [2, 3]. However, the reperfusion process can lead to paradoxical damage known as ischemia–reperfusion (IR) injury. IR injury can trigger rhabdomyolysis, skeletal muscle necrosis, electrolyte imbalances, acute renal injury, and, in severe cases, even death [4–6]. At present, no effective measure is available to treat the patient after fasciotomy. The previous studies have indicated that a significant number of systemic complications, ranging from 15 to 25%, can occur after fasciotomy in patients with ACS [7]. Furthermore, approximately 30–40% of these patients may experience long-term limb function defects [8–10]. Thus, it is imperative to delve into efficacious therapeutic modalities for ACS.

The present studies suggest that IR-induced inflammation plays a significant role in ACS [1, 11]. ACS leads to reduced blood perfusion and deterioration and expeditious leukocyte activation in skeletal muscle tissue [12]. The aggregation of inflammatory cells adhering to venules engenders direct harm to capillary perfusion, promoting augmented vascular protein leakage and edema. Subsequent to fasciotomy, the inflammatory response and reperfusion-related skeletal injury burgeon even further alongside the rise in tissue perfusion [4, 13, 14]. In this intricate process, macrophages play a important role in the regulation of skeletal muscle inflammation. Following the reperfusion phase, M1 macrophages generate a pro-inflammatory environment and clear cellular debris. Gradually, the infiltration of M2 macrophages increased. M2 macrophages not only secrete anti-inflammatory cytokines but also release a diverse array of growth factors, exerting a crucial influence on skeletal muscle healing and scar formation [15, 16]. Thus, both of these distinct phenotypes hold significant importance in the healing of skeletal muscle. The alteration in balance between M1 and M2 macrophages, resulting in earlier and more abundant M2 macrophage infiltration, offers a potential target for the treatment of muscle I/R injury.

Our previous study induced phenotype-consistent embryonic stem cells-derived mesenchymal stem cells (ESC–MSCs) and verified that ESC–MSC therapy could alleviate ACS-induced severe skeletal muscle injury and foster the mending of muscular tissue upon injury through regulating macrophage polarization [17]. Due to their capacity for large-scale amplification and phenotypic homogeneity in comparison with regular MSC populations, ESC–MSCs and their derivatives possess significant advantages in clinical application [18, 19]. Extracellular vesicles (EVs) are regarded as secretory components that contain a unique assortment of small RNAs and proteins, and play a pivotal role in the physiological impacts facilitated by ESC–MSCs. Compared to stem cell transplantation, EV therapy represents a more advantageous choice for clinical applications due to its benefits, including reduced immunogenicity, heightened safety, convenient storage and handling, and the ability for mass production [20]. To date, no studies have investigated the effects of ESC–MSC-EVs in diseases. The therapeutic functions and mechanisms of ESC–MSC-EVs in ACS also remain unknown.

Here, we investigated the therapeutic capabilities of ESC–MSC-EVs on the ACS model. Remarkably, ESC–MSC-EVs exhibited substantial efficacy in mitigating skeletal muscle injury, stimulating angiogenesis, and facilitating the recovery of skeletal muscle function in ACS rats. This was achieved through the targeted delivery of diverse miRNAs to macrophages. Furthermore, we identified potential miRNA candidates responsible for regulating macrophage polarization.

## Material and methods

### Animal studies

Ethical approval for the animal experiments outlined in this study was granted by the Institutional Ethics Committee of the Second Affiliated Hospital of Zhejiang University School of Medicine. The manuscript adheres to the ARRIVE guidelines for the reporting of animal experiments. Ninety-six male Sprague–Dawley rats, approximately 60 d old and weighing approximately 250 ± 10 g, were obtained from SLAC Laboratory Animal Co., Ltd. located in Shanghai, China. Animals were randomly distributed in the cages of the different groups. The rodents were kept in a regulated setting with a temperature of 22 ± 1 °C, while the air moisture was maintained at 60 ± 5%. Additionally, they were exposed to a 12-h cycle alternating between light and darkness. The animals were given unlimited access to food and water to ensure that they had unrestricted availability.

The ACS model was created as we previously outlined [17]. Briefly, the rats were anesthetized through an intraperitoneal injection of 3% pentobarbital sodium. After the rats were unconscious, a 24-gauge angiocatheter was inserted into the anterior compartment of the left hindlimb in the experimental group to increase the pressure. The pressure was raised to 80 mmHg and kept at 80 ± 10 mmHg for 2 h. A single-incision fasciotomy was performed at the end of the experiment to relieve the pressure in the compartment. In the control group, the rats underwent all procedures except the pressure was maintained at the baseline level (0 mmHg).

We utilized the NC3Rs website to ascertain the optimal and adequate number of rats per group required to attain statistically meaningful outcomes. During the reception of the animals, the attainment of traceability and individual identification was established through the utilization of ear pins. The researchers possessed awareness throughout the process of allocation, the execution of the experiment, the assessment of outcomes, and the analysis of data according to the ARRIVE guidelines. To evaluate the treatment using ESC–MSC-EVs, the experimental groups were then divided into four groups of six rats each: Sham + vehicle group, Sham + EVs group, ACS + vehicle group, and ACS + EVs group by means of the random number method. To treat the rats, either ESC–MSC-EVs (100 μg) or a control vehicle (PBS) were given through the dorsal penis vein. At the conclusion of the trial, the rodents were sacrificed by administering an excessive amount of 3% pentobarbital sodium through an intra-arterial injection. Every effort was made to minimize suffering by adding enrichment such as excessive anesthesia and rapid execution. Samples of blood and muscle from the tibialis anterior (TA) were gathered for additional examination.

### ESC–MSCs preparation and identification

A two-step process was used to differentiate ESCs into ESC–MSCs as we have previously described [17]. Flow cytometry was utilized to phenotypically characterize ESC–MSCs by employing specific antibodies obtained from BD Pharmingen (USA). The antibodies used for this analysis included anti-CD31, anti-CD90, anti-CD105, anti-CD19, anti-CD45, anti-CD14, anti-CD34, anti-CD11b, anti-CD79a, and anti-HLA-DR.

Multi-lineage differentiation potency of ESC–MSCs was evaluated by inducing osteogenic, adipogenic, and chondrogenic differentiation. All differentiation media and dyes are purchased from Cyagen (China). For osteogenic differentiation, ESC–MSCs (2 × 10^4^ cells/cm^2^) were cultured in 6-well plates coated with 1% gelatin and grown until reaching 60–70% confluency. The medium for promoting the formation of osteogenic differentiation was changed every 3 d. After a 3-w induction period, calcium nodules were identified by staining with alizarin red. For chondrogenic differentiation, 5 × 10^5^ was formed through centrifugation at 1000 rpm for 5 min. The pellet was then cultured in 15-mL centrifuge tubes containing chondrogenic differentiation medium for a duration of 3 w. After 4% paraformaldehyde fixation and frozen sections, alcian blue is used to dye frozen sections. Adipogenic induction commenced upon the full confluence of the cell culture. After being isolated, the cells were cultured in adipogenic differential medium A for 3 d. Following this initial phase, the cells were then transitioned to adipogenic differential medium B for a single day of maintenance. This dynamic process was repeated for a total of 6 cycles to promote the synthesis and accumulation of lipid droplets within the cells. Subsequently, the cultures underwent fixation utilizing the 4% paraformaldehyde solution, followed by staining using oil red O.

### Extracellular vesicles (EVs) isolation and identification

ESC–MSCs were cultured using a serum-free MSC proliferation medium (ExCell Bio, China). EVs were extracted using ultracentrifugation previously described [21]. Briefly, the supernatant of the P4 generation ESC–MSCs cell culture was collected and subjected to centrifugation at 4 °C and 300 g for 5 min. To eliminate any remaining cells and cell fragments, the centrifugation was extended for 15 min at 3000*g* (4 °C). After filtration, an ultra-high-speed centrifuge (Beckman, USA) was used to centrifuge at 4 °C and 10,000 *g* for 30 min to remove cellular organelles, followed by centrifugation at 4 °C and 120,000 *g* for 70 min to obtain EVs. A suitable amount of PBS solution was then added to resuspend.

The procedural method for observing samples using transmission electron microscopy (TEM) is as follows: A volume of 10 μL of the sample was dispensed onto a copper mesh, subsequently permitting sedimentation for 1 min, followed by absorption of the supernatant using filter paper. Subsequently, a volume of 10 μL of a 4% uranyl acetate solution was added to the copper mesh, allowing it to settle for 10 min, after which the supernatant was once more absorbed using filter paper. After several min of air-drying at room temperature, the imaging was inspected using an electron microscope (JEOL, Japan) at 120 kV.

The particle size analysis procedure is as follows: 30 μL of the sample was taken, and the analysis of particle size was conducted employing NanoSight NS500 (Malvern Instruments, UK).

Western blotting was used to further examine the surface characteristic proteins Calnexin, HSP70, TSG101, CD63, and CD9.

### Pathological analysis

Paraffin sections with a thickness of 4 μm were prepared and subsequently subjected to staining using either H and E or Masson trichrome methods. The stained sections were analyzed using a light microscope (Leica, Germany) at 200× magnification. The determination of the proportion of injured myofibers was conducted following the protocol outlined by McCormack et al. [22]. Injured myofibers are defined as ragged cellular edges, vacuolation, lymphocyte infiltration, or rhabdomyolysis. Myofibers that contain central nuclei were identified as regenerative [23]. Regenerated myofibers were calculated as described in our previous study [17]. Briefly, for each sample, five random fields were chosen and observed using a light microscope (Leica) at 200× magnification. The aim was to determine the total count of regenerative myofibers. Additionally, the minor axis diameters of these fibers in each TA muscle were measured with the assistance of ImageJ software (NIH, USA). In order to evaluate the fibrotic region in sections of skeletal muscle, five random fields were chosen from each sample using a light microscope (Leica) at 200× magnification. The percentage of the fibrotic area was determined utilizing ImageJ software.

### TUNEL and dystrophin staining analysis

Skeletal muscle apoptosis was measured by performing TUNEL and dystrophin immunofluorescence staining on muscle tissue frozen sections (10 μm). The TUNEL staining was carried out following the protocol of the manufacturer (Roche Inc., Switzerland). Subsequently, the sections were subjected to an incubation step with anti-dystrophin (1:200, 12715-1-AP, Proteintech) and succeeded by an anti-rabbit IgG cyanin 3 (Cy3) secondary antibody (1:200, SA00009-2, Proteintech). DAPI (Meilunbio, China) was used for counterstaining the nuclei. The sections were analyzed using an upright fluorescence microscope (Olympus, Japan) at 200× magnification. The photomicrographs were combined using the Image-Pro Plus software (Olympus). The quantity of TUNEL-positive and DAPI-positive nuclei was enumerated, taking into account solely those labeled nuclei which exhibited co-localization with dystrophin staining. The findings were articulated in the form of the TUNEL index, acquired by dividing the tally of TUNEL-positive nuclei by the aggregate number of nuclei. The calculation of the TUNEL index was performed for each section by scrutinizing five random and non-overlapping fields.

### Western blotting

Protein extraction was performed from EVs, cells, or tissues using a protein lysis buffer containing PMSF (Biosharp, China). For the detection of phosphorylated proteins, an additional phosphatase inhibitor (Biosharp) was added to the lysis buffer. The quantification of proteins was accomplished through the utilization of a BCA kit (Biosharp), while their separation was performed via sodium dodecyl sulfate–polyacrylamide gel electrophoresis, followed by their transfer onto PVDF membranes. Subsequently, the PVDF membranes underwent a blocking process utilizing a solution composed of 5% skimmed milk in Tris-buffered saline. In the subsequent step, the membranes were subjected to overnight incubation at 4 °C with primary antibodies. Primary antibodies that we used include anti-Calnexin (abcam, ab133615), anti-HSP70 (abcam, ab181606), anti-TSG101 (abcam, ab125011), anti-CD63 (abcam, ab134045), anti-CD9 (abcam, ab263019), anti-β-actin (CST, #4970S), anti-iNOS (Invitrogen, #PA3-030A), anti-Bcl-2 (CST, #3498S), anti-Bax (CST, #14796S), anti-Cleaved caspase 3 (CST, #9664S), anti-CD206 (CST, #24595), anti-CD68 (abcam, ab283654), anti-CD86 (abcam, ab220188), anti-Arg1 (CST, #93668S), anti-P65 (CST, #8242S), anti-phospho-P65 (CST, #3033S), anti-IKBα (CST, #4812S), anti-phospho-IKBα (CST, #2859S), anti-JAK1 (CST, #3344T), anti-phospho-JAK1 (CST, #74129S), anti-STAT6 (CST, #5397S), anti-phospho-STAT6 (CST, #56554S), anti-STAT3 (CST, #12640S), anti-phospho-STAT3 (CST, #9145S), anti-PI3K (CST, #4249T), anti-phospho-PI3K (CST, #13857S), anti-AKT (CST, #9272S), anti-phospho-AKT (CST, #4060T), anti-PTEN (Biolegend, USA, 4C11A11), anti-NLRP3 (CST, #15101S), anti-mTOR (CST, #2983T), and anti-TLR3 (CST, #6961S). Next, the PVDF membranes were exposed to the appropriate HRP-linked antibodies and incubated at room temperature for a duration of 2 h. The bands were made visible through the use of an ECL kit (Millipore, USA) and measured by ImageJ software.

### Serum and cell culture supernatants analysis

In this study, serum was obtained by collecting blood samples from the abdominal aorta. Creatine kinase was measured in samples using an LW C400 Clinical Chemistry Analyzer (Landwind Medical, China). Additionally, ELISA kits were employed to measure the concentrations of TNF-α, IL-6, and IL-10 (Elabscience, China) in both the serum and cell culture supernatant. The samples were evaluated for OD at a wavelength of 450 nm using a microplate reader (Thermo Fisher Scientific, USA).

### Rat ethology

In this study, skeletal muscle function was assessed using the hanging grid test and grip strength test. The hanging grid test involved placing rats individually at the center of a wire mesh screen suspended 50 cm above a plastic cage filled with sawdust bedding. The hanging duration was recorded in three independent trials, and the data from all three trials were averaged to obtain a final value. This test is commonly used to evaluate muscle strength and endurance in rodents [17, 24].

The grip strength test was utilized in this study to assess muscle strength in rats. The test was conducted using a grip strength meter (Handpi HP-5N, China). During the test, the rats were held by the tail and approached the grid slowly until their hind claws grasped the grid. They were then gently pulled by the tail until they released their grip, and the forces of three trials were recorded and averaged to obtain the final value. This test is commonly used to evaluate muscle strength and has been previously described [17, 25].

### Immunofluorescence staining

The paraffin-embedded muscle tissue sections (4 μm) were utilized. These sections were subjected to antigen retrieval, blocking, and labeling with primary antibodies against CD86 (Santa Cruz, sc-20060) and CD206 (Santa Cruz, sc-58986), followed by staining with FITC- or Cy3-labeled secondary antibodies. Negative controls were included in the experiment to ensure the specificity of the staining. After the staining process, the sections were observed using an upright fluorescence microscope (Olympus) at 200× magnification. Immunofluorescent staining of CD31 (R and D Systems, USA, AF3628) was performed on muscle tissue frozen sections (10 μm) using the method described above.

### Quantitative real‑time PCR

The extraction of RNA was carried out by utilizing TRIzol reagent (Invitrogen, USA) according to the instructions provided by the manufacturer. To determine the concentration and purity of the RNA sample, the Nanodrop spectrophotometer (Thermo Fisher) was used for quantifying the extracted RNA. After extracting RNA, the PrimeScript RT reagent kit (Yeason, China) was utilized to perform reverse transcription, converting RNA into cDNA. The Mx3000P real-time PCR system (Agilent Technologies, USA) was used to perform quantitative real-time PCR with SYBR Mixture (Yeason), specific primers (as indicated in Additional file 1: Table S1), and the cDNA samples. For normalization purposes, the internal reference gene β-actin was employed in housekeeping.

### Macrophages depletion

One ml of liposomal clodronate (LC, 5 mg/ml, Vrije Universiteit, The Netherlands) was injected into the rats via the intravenous route. LC administration was conducted 1 d prior and 1 d following the induction of ACS injury, as previously explained [17, 26]. This method has been previously described in detail. Rats were injected with liposomal vehicle (LV, Vrije Universiteit) as the control.

### Immunohistochemistry

The paraffin-embedded muscle tissue sections (4 μm) were utilized. These sections were subjected to antigen retrieval, blocked, and labeled overnight at 4 °C using anti-CD68 (1:200, Santa Cruz, sc-20060). HRP-labeled secondary antibodies (Boster, China) were used, followed by the addition of an avidin–biotin–peroxidase conjugate (Boster). The resulting response was observed using a diaminobenzidine (DAB) chromogen solution for substrate (VectorLabs, USA). In the end, the parts were stained with hematoxylin and examined using light microscopy (Leica) at 200× magnification.

### Macrophage differentiation and stimulation

The THP-1 cells were grown in a specialized solution (Procell, China) with a cell density ranging from 3 × 10^5^ to  6 × 10^5^ cells/ml. THP-1 cells were subjected to a treatment of phorbol 12-myristate 13-acetate (PMA, 160 ng/ml, Sigma, USA) for 24 h to induce a macrophage-like phenotype. Subsequently, the adherent cells were cultivated in a fresh medium supplemented with LPS (100 ng/ml, Sigma) and IFN-γ (50 ng/ml, Novoprotein, China). Following this, the conditioned medium was substituted with fresh medium alone or in combination with ESC–MSC-EVs (10 μg/ml) for an additional 48 h.

### EVs labeling and cellular uptake assay

EVs were labeled with PKH67 (Sigma) for 2 min at room temperature. Addition of PKH67 to PBS solution without EVs as the control. The labeled EVs suspension was filtered with a Spin Column (Invitrogen, USA) to remove unbound dye according to the previous study [27]. THP-1 cells (1 × 10^6^) were grown in a 6-well dish and then exposed to PKH67-labeled EVs (10 ug/ml) for a duration of 6 h. The cells were subsequently treated with DAPI (Meilunbio) and TRITC-conjugated phalloidin (Yeason) and examined using an inverted fluorescence microscope (Olympus) at 400× magnification.

### Flow cytometry analysis

TrypLE™ Express trypsin (Biosharp, China) was used to detach the cells from the plate. In order to prevent non-specific binding, 100 μL of a 10% solution of Gamunex (a solution of human immune globulin) was introduced and left to incubate on ice for a duration of 10 min. This was followed by incubation with anti-CD206 (#321103, Biolegend, USA) and anti-CD86 (#374205, Biolegend) antibodies for 30 min in the dark. After centrifugation, cells were washed twice and immediately measured by the flow cytometer (Beckman), and data evaluation was performed using CytExpert 2.4 software (Beckman).

### Transcriptome sequencing and bioinformatic analysis

THP-1 cells were differentiated and stimulated, then cultured in either a normal medium or medium containing ESC–MSC-EVs for 48 h. The sequencing was performed by LC-BIO (China), and the methodology was as previously described [28]. Briefly, Illumina Novaseq 6000 (USA) was utilized for paired-end sequencing in PE150 sequencing mode, following standard procedures. During the analysis phase, CleanData was aligned to the Homo sapiens genome using HISAT2 software. mRNA expression levels were subsequently analyzed by both StringTie and Ballgown software packages. The R package was employed to analyze differences in mRNA expression levels between the two groups. Genes with a different fold change greater than 2, or less than 0.5, and a p value less than 0.05 were selected as differential genes. The mRNA exhibiting differential expression was subsequently subjected to enrichment analysis based on GO and KEGG.

### Small RNA sequencing

LC-BIO conducted the analysis of small RNA sequencing using the previously described methodology [29]. Briefly, ESC–MSC-EVs were used to extract total RNA with the mirVana miRNA isolation kit (Ambion). Small RNA libraries were constructed using TruSeq Small RNA Sample Prep Kits (Illumina) with 1 μg of total RNA from each sample. The libraries were sequenced on an Illumina HiSeq X Ten platform after PCR amplification and size selection. Differentially expressed miRNAs were identified with a *p* value threshold of < 0.05.

### MicroRNA transfection

The miRNA transfection was performed as previously described [30]. Chemically synthesized and modified miRNA mimics (Additional file 1: Table S1) or mimics NC from Sangon Company (China) were transfected into THP-1 cells using the FuGENE HD transfection reagent (Promega, USA) following the instructions provided by the manufacturer.

### Statistical analysis

GraphPad Prism 8.0 (USA) was utilized for both statistical and graphical analyses in this study. The data were expressed in terms of mean and standard deviation (SD). Two-group comparisons were analyzed using Student’s t-tests, whereas multiple group comparisons were performed using one-way ANOVA with Tukey’s post hoc test and two-factor ANOVAs with Bonferroni pairwise comparisons. Statistical significance was considered at a *p* value < 0.05.

## Results

### Characteristics of ESC–MSCs

ESC–MSCs obtained from ESCs differentiation developed a populace of cells exhibiting a uniform phenotype characterized by spindle-shaped morphology. Figure S1A visually depicts the morphological characteristics of ESC–MSCs at both P0 and P4. Additional file 1: Fig. S1B provides visual evidence of the expression of MSC markers. Notably, the proportions of cells positive for CD73, CD90, and CD105 were determined to be 99.42%, 99.36%, and 97.99%, respectively. Conversely, ESC–MSCs exhibited negligible expression (less than 1%) of CD19, CD45, CD14, CD34, CD11b, CD79a, and HLA-DR, affirming their negative phenotype. The multipotency of ESC–MSCs was demonstrated by inducing cells to differentiate along the lineage of osteogenic, chondrogenic, and adipogenic (Additional file 1: Fig. S1C).

### Isolation and characterization of EVs

**Fig. 1:**
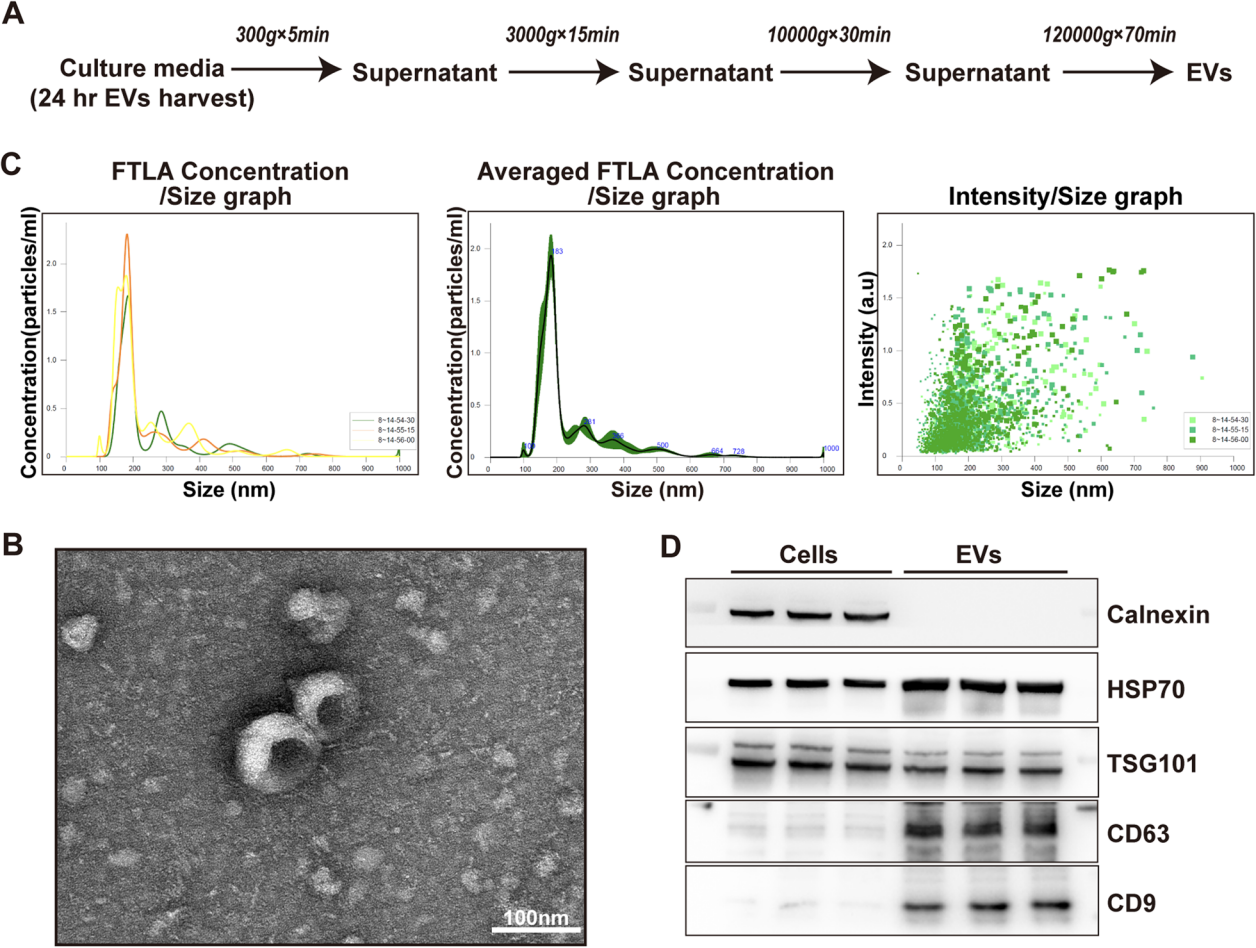
Isolation and characterization of ESC–MSC-EVs. **A** Flowchart for the ESC–MSC-EVs isolation and purification process based on differential ultracentrifugation. **B** Representative image of ESC–MSC-EVs captured using TEM. Scale bar: 100 nm. **C** ESC–MSC-EVs were analyzed using NTA. D The expression of Calnexin, HSP70, TSG101, CD63, and CD9 in ESC–MSCs and ESC–MSC-EVs was determined by Western blotting. The blots of **D** were all cropped, and full-length blots were presented in Additional file 2: Fig. S1

Figure 1A illustrates the procedure employed for the isolation of ESC–MSC-EVs via ultracentrifugation. The characterization involved assessments of morphology, size distribution, and surface markers of the ESC–MSC-EVs [31] (Fig. 1B–D). The EVs secreted by ESC–MSCs exhibited a bilayer structure and a circular shape (Fig. 1B). The results from the Western blotting indicated significant enrichment of four surface marker proteins (HSP70, TSG101, CD63, and CD9), and Calnexin was not expressed in EVs samples relative to equivalent protein concentrations from ESC–MSCs (Fig. 1C).

### ESC–MSC-EVs decrease skeletal muscle injury and systemic inflammation after ACS

**Fig. 2:**
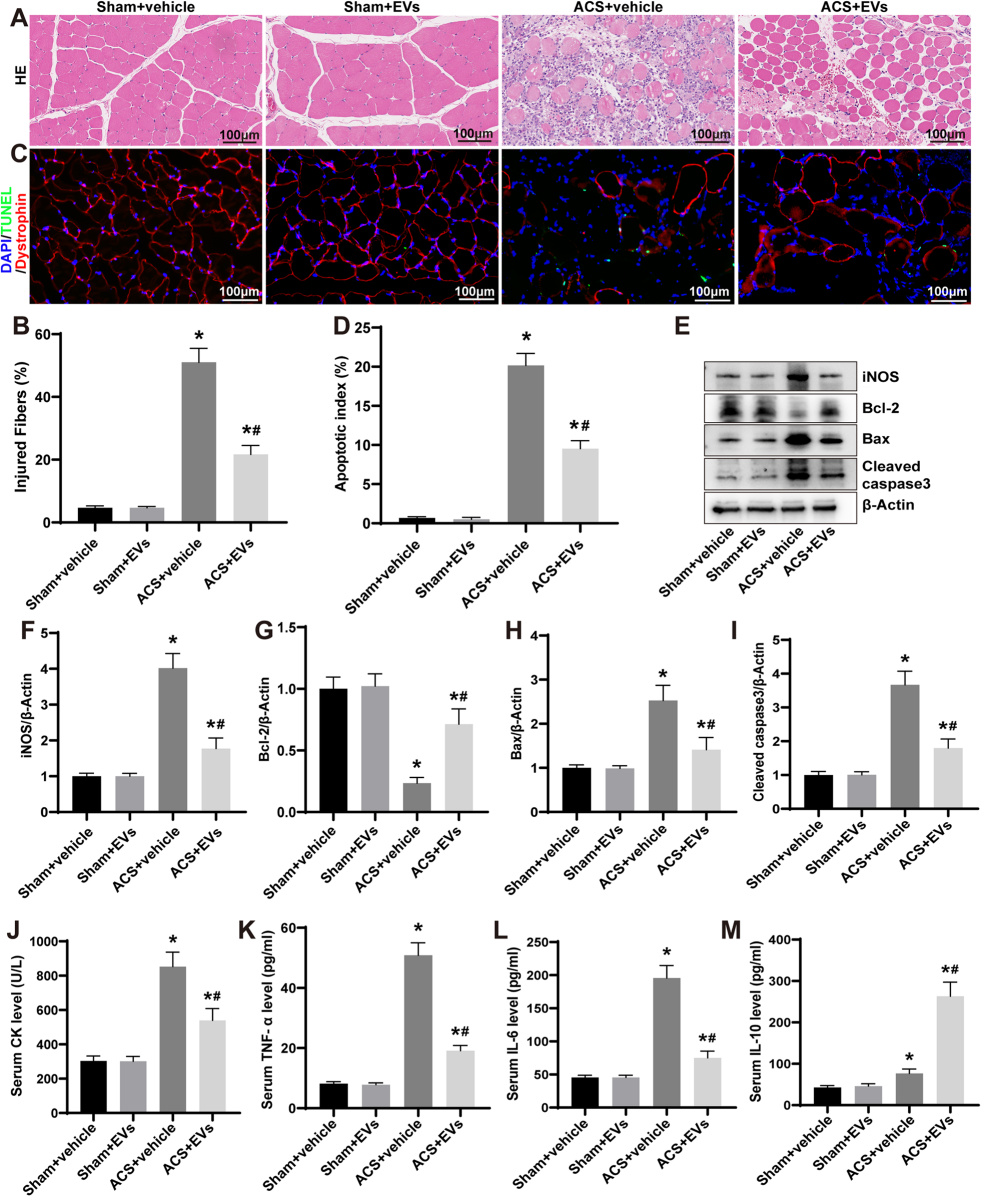
The impact of ESC–MSC-EVs on skeletal muscle injury and systemic inflammation in ACS rats. **A** Representative images (200×) of HE staining of TA muscle sections at 3 d after ACS. Scale bar: 100 μm. **B** Percentage of injured myofibers (*n* = 6). **C** Representative images (200x) of TUNEL staining of TA muscle sections at 3 d after ACS. Scale bar: 100 μm. **D** Percentage of apoptotic skeletal muscle fibers nuclei (*n* = 6). **E** Representative images of iNOS, Bcl-2, Bax, and Cleaved caspase 3 expression levels of skeletal muscle by Western blotting at 3 d after ACS. **F**–**I** Quantitative analysis expression of iNOS, Bcl-2, Bax, and Cleaved caspase 3 (*n* = 6). **J** Serum CK levels at 3 d after ACS (*n* = 6). **K**–**M** Serum TNF-α, IL-6, and IL-10 levels at 3 d after ACS (*n* = 6). Data are presented as the mean ± SD. *P < *0.05* versus Sham + vehicle group and ^***#***^*P* < *0.05* versus ACS + vehicle group. The blots of **E** were all cropped, and full-length blots were presented in Additional file 2: Fig. S2

Severely disorganized and degenerated myofibers were observed in ACS rats at 3 d, and the proportion of injured myofibers was as high as 51 ± 4.45% (Fig. 2A and B). In contrast, these unfortunate changes in skeletal muscle tissue were ameliorated by ESC‑MSCs-EVs treatment, and the proportion of injured myofibers decreased to 21.67 ± 2.83% (Fig. 2A and B). Apoptosis of skeletal muscle cells was detected using TUNEL staining, which labels fragmented DNA in apoptotic cells, while dystrophin immunofluorescence staining was used to identify skeletal muscle cells. In this analysis, TUNEL-positive apoptotic nuclei were observed to co-stain with dystrophin, indicating the apoptotic skeletal muscle cells (Fig. 2C; Additional file 1: Fig. S2). The apoptotic index in the ACS + vehicle group was 20.15 ± 1.54%, while the ACS + EVs group was significantly reduced to 9.53 ± 1.02% (Fig. 2D). Furthermore, the expression of the inflammatory protein iNOS, the proapoptotic protein Bax and Cleaved caspase 3, and the antiapoptotic protein Bcl-2 in skeletal muscle tissue was measured (Fig. 2E). ESC–MSC-EVs showed a remarkable reduction of iNOS, Bax, and Cleaved caspase3, and an increase in Bcl-2 in ACS rats (Fig. 2F–I). Moreover, serum analysis suggested that ESC‑MSCs-EVs treatment reduces serum CK level, a marker of skeletal muscle damage, in ACS rats (Fig. 2J), and reduced the mRNA expression of TNF-α and IL-6, while increasing the mRNA expression of IL-10 (Fig. 2K–M). Based on the data, it can be inferred that treatment with ESC–MSC-EVs has a beneficial effect in mitigating skeletal muscle injury and systemic inflammation induced by ACS.

### ESC–MSC-EVs facilitate the regeneration of myofibers, diminish tissue fibrosis, and expedite the restoration of muscle function following ACS

**Fig. 3:**
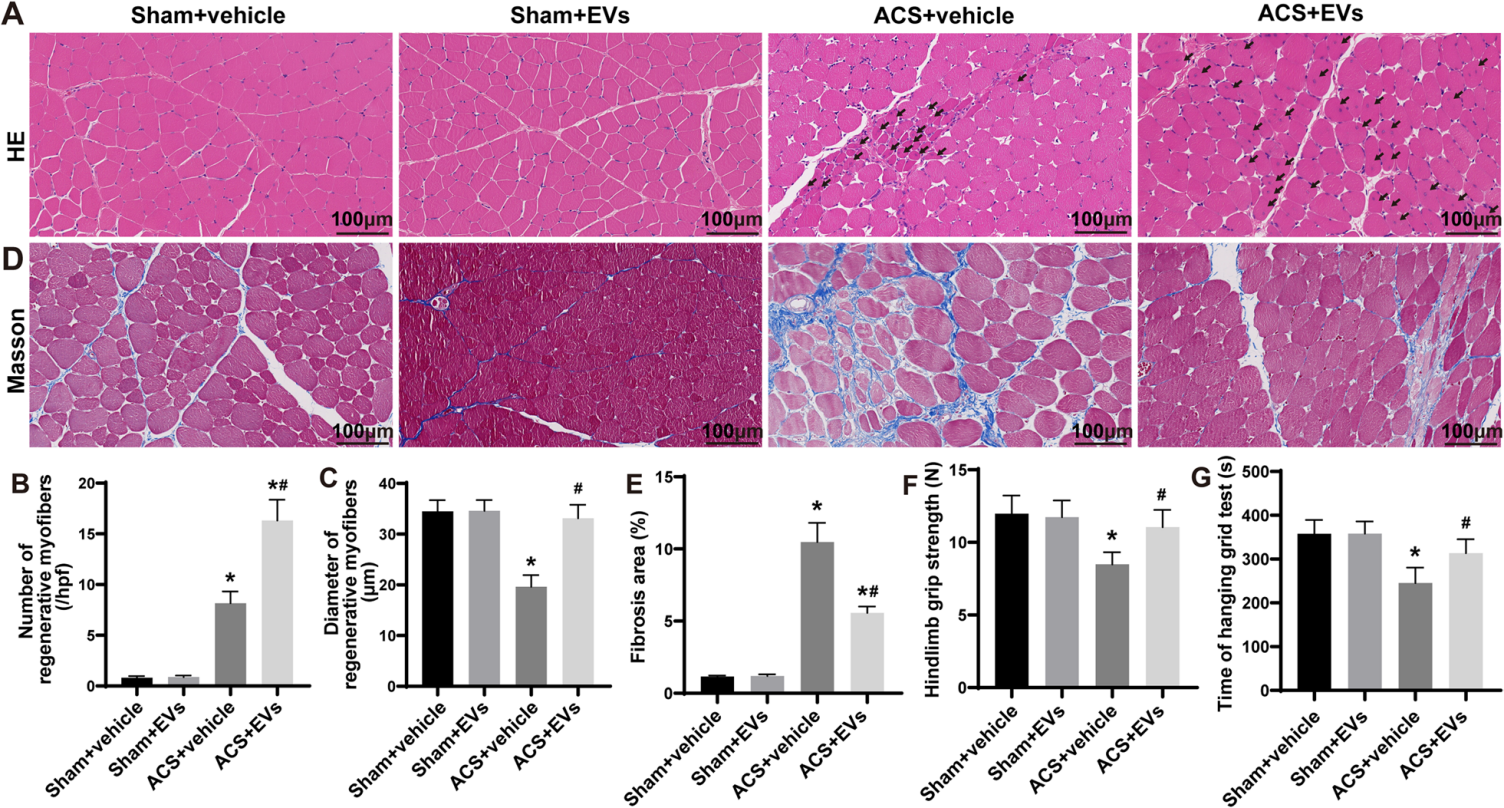
The impact of ESC–MSC-EVs on skeletal muscle fiber regeneration, tissue fibrosis, and muscle function in ACS rat. **A** Representative images (200×) of HE staining of TA muscle sections at 7 d after ACS (myofibers that contained a central nucleus were used as the definition for regenerating myofibers). Scale bar: 100 μm. **B** Quantitative analysis pertaining to the numerical quantity of rejuvenating myofibers (*n* = 6). **C** Quantitative appraisal concerning the width of regenerating myofibers (*n* = 6). **D** Representative images (200x) of Masson staining of TA muscle sections at 14 d after ACS. Scale bar: 100 μm. **E** Quantitative analysis of the fibrotic area of skeletal muscle tissue sections at 14 d after ACS (*n* = 6).** F** Grip strength at 14 d after ACS (*n* = 6). **G** Hanging time at 14 d after ACS (*n* = 6). Data are presented as the mean ± SD. *P < *0.05* versus Sham + vehicle group and ^***#***^*P* < *0.05* versus ACS + vehicle group

Skeletal muscle tissue sections on 7 d with HE staining show little regenerating myofibers observed in the Sham groups (Fig. 3A–C). The regenerated myofibers with shorter diameters were increased in the ACS + vehicle group (Fig. 3A–C). In contrast, the ACS + EVs group exhibited a substantial increase in both the quantity and diameter of regenerating myofibers (Fig. 3A–C). Subsequently, we conducted an observation of the fibrosis of skeletal muscle tissue 14 d after ACS. The skeletal muscle tissue sections obtained on the 14 d were subjected to Masson staining, which revealed that ACS resulted in severe fibrosis of skeletal muscle tissue. However, this fibrosis was observed to be reduced following treatment with ESC–MSC-EVs (Fig. 3D–E). Moreover, muscle function evaluation of 14 d suggested that the rats in ACS + vehicle group experienced a decline in grip strength of the hindlimb as well as a reduction in the hang time on a wire mesh screen, while ESC‑MSC-EVs treatment significantly enhanced muscle function (Fig. 3F–G). These results reveal that ESC‑MSC-EVs promote tissue repair, reduce tissue fibrosis, and facilitate the recovery of muscle function after ACS.

### ESC–MSCs-EVs promote the polarization of M2 macrophages and the process of angiogenesis within skeletal muscle tissue subsequent to ACS

**Fig. 4:**
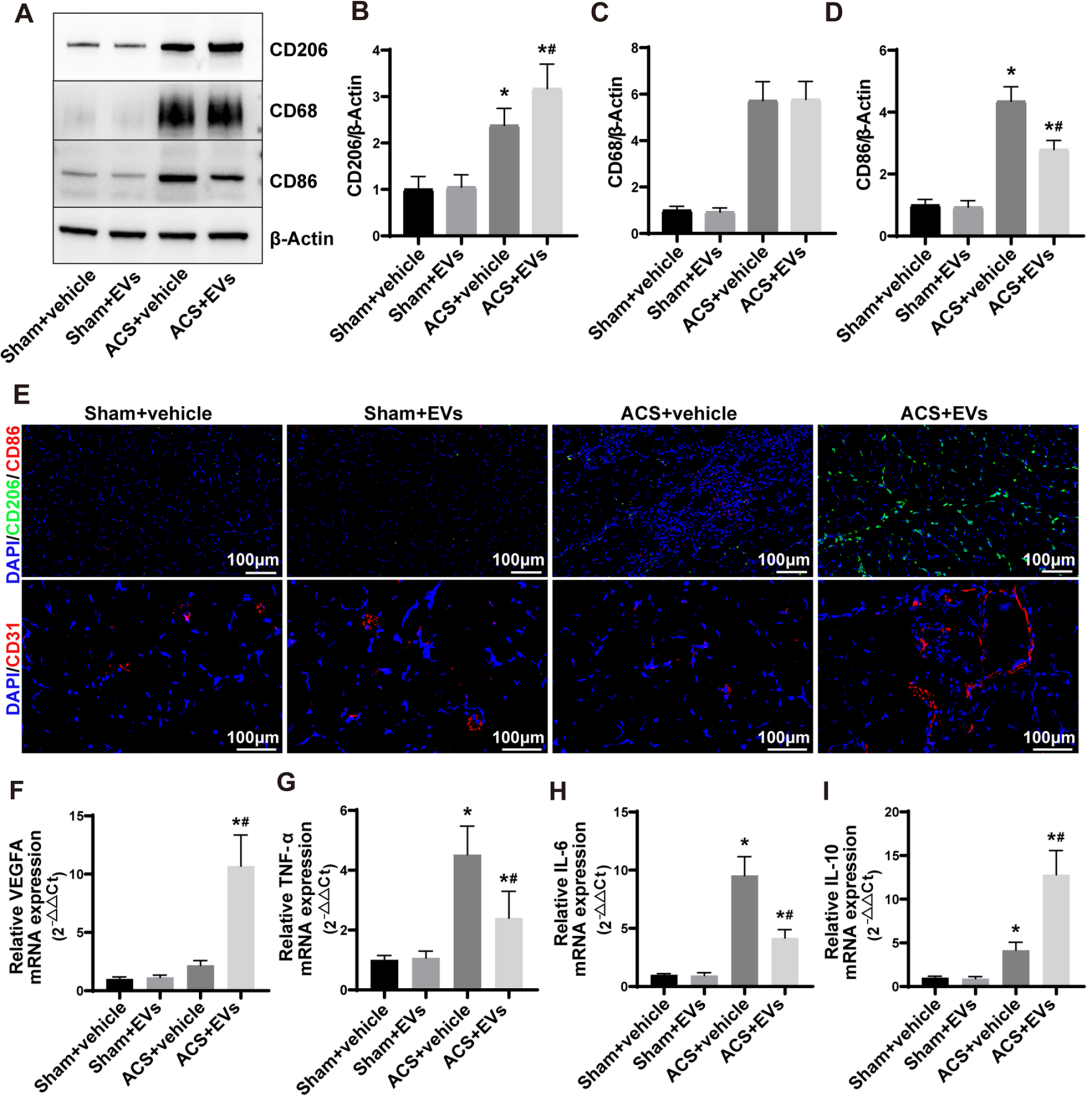
The impact of ESC–MSC-EVs on the polarization of macrophages and angiogenesis in the skeletal muscle tissue of ACS rats. **A** Representative images of CD206 (M2 macrophage marker), CD68 (macrophage marker), and CD86 (M1 macrophage marker) expression levels of skeletal muscle by Western blotting at 3 d after ACS. **B**–**D** Quantitative analysis expression of CD206, CD68, and CD86 (*n* = 6). **E** Representative images (200×) depicting immunofluorescent staining for CD206, CD86, and CD31 on muscle tissue sections. Scale bar: 100 μm. **F** The gene expression of VEGFA in skeletal muscle tissue at 3 d after ACS was measured via RT-qPCR (*n* = 6). **G**–**I** The gene expression of TNF-α, IL-6, and IL-10 in skeletal muscle tissue at 3 d after ACS was measured via RT-qPCR (*n* = 6). Data are presented as the mean ± SD. *P < *0.05* versus Sham + vehicle group and ^***#***^*P* < *0.05* versus ACS + vehicle group. The blots of **A** were all cropped, and full-length blots were presented in Additional file 2: Fig. S3

To investigate the role of macrophages in the beneficial effects of ESC–MSC-EVs on ACS, we first assessed the expression of CD206, CD68, and CD86 in skeletal muscle tissue subjected to ESC–MSC-EVs intervention on 3 d (Fig. 4A). After the occurrence of ACS, there is a notable augmentation in the manifestation of the CD68 protein, associated with macrophages, within muscular tissue, along with an upsurge in the expression of the CD86 protein, characteristic of M1 macrophages. Conversely, treatment involving ESC–MSC-EVs showcases a reduction in the expression of CD86 protein, while concurrently enhancing the expression of CD206 protein in the skeletal muscle tissue. However, there was no statistical difference in the expression of CD68 in muscle tissue between the ACS groups (Fig. 4B–D). In addition, immunofluorescence staining revealed an escalation in M1 (CD86 +) macrophage infiltration within the ACS + vehicle group's muscle tissue, whereas M1 macrophage infiltration receded and M2 macrophage infiltration appreciably intensified in the ACS + EVs group. Moreover, ESC‑MSCs-EVs treatment significantly promoted neovascularization (CD31 +) at 3 d after ACS (Fig. 4E; Additional file 1: Fig. S3A-B) and upregulated the mRNA expression of pro-angiogenic factors VEGFA (Fig. 4F). The mRNA expression of pro-inflammatory cytokines such as TNF-α and IL-6 was significantly increased at 3 d after ACS. ESC‑MSC-EVs treatment downregulated the mRNA expression of TNF-α and IL-6 and upregulated mRNA expression anti-inflammatory cytokine IL-10 (Fig. 4G–I). The data presented suggest that ESC–MSC-EVs treatment results in a notable reduction in the inflammatory response and a consequential increase in angiogenesis in the ACS-induced skeletal muscle injury model. This is achieved through the induction of M2 macrophage polarization.

### The effectiveness of ESC–MSC-EVs treatment was decreased by the depletion of macrophages

**Fig. 5:**
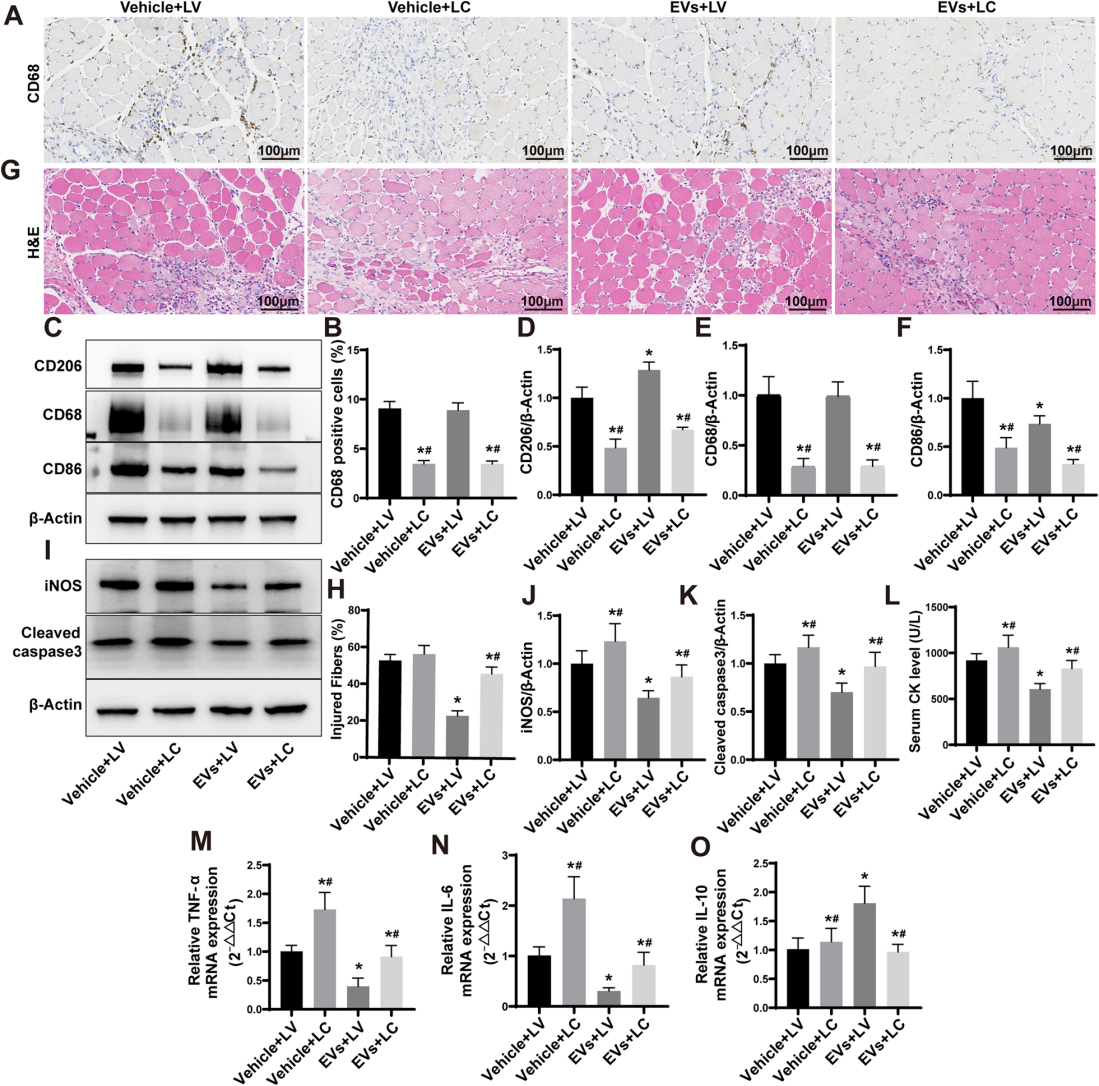
The impact of macrophage depletion on ESC–MSC-EVs therapy in severe skeletal muscle injury caused by ACS. **A** Representative immunohistochemistry images (200**x**) of TA muscle sections stained with CD68 at 3 d after ACS. Scale bar: 100 μm. **B** Quantitative assessment of cells positive for CD68 (*n* = 6). **C** Representative images of CD206, CD68, and CD86 expression levels of skeletal muscle by Western blotting at 3 d after ACS. **D**–**F** Quantitative analysis expression of CD206, CD68, and CD86 (*n* = 6). **G** Representative images (200×) of HE staining of TA muscle sections at 3 d after ACS. Scale bar: 100 μm. **H** Percentage of injured myofibers (*n* = 6). **I** Representative Western blotting images of iNOS and Cleaved caspase 3 expression levels of skeletal muscle at 3 d. **J**–**K** Quantitative analysis expression of iNOS and Cleaved caspase 3 (n = 6). **L** Serum CK levels at 3 d (*n* = 6). **M**–**O** The mRNA expression of TNF-α, IL-6, and IL-10 in skeletal muscle tissue at 3 d after ACS (*n* = 6). Data are presented as the mean ± SD. *P < *0.05* versus Sham + vehicle group and ^**#**^*P* < *0.05* versus ACS + vehicle group. The blots of Additional file 2: Fig. S5C and I were all cropped, and full-length blots were presented in Additional file 2: Figs. S4 and S5

In order to delve deeper into the contribution of macrophages toward the healing properties of ESC–MSC-EVs, liposomal clodronate (LC, macrophage scavenge) was administered via injection into ESC–MSC-EVs treated ACS rats to deplete macrophages. Immunohistochemical analysis revealed a noteworthy reduction in the expression of the macrophage marker CD68 within the skeletal muscle tissue at 3 d (Fig. 5A and B). Additionally, utilizing Western blotting, it was confirmed that depleting macrophages resulted in a decrease in the expression of macrophage-associated proteins within the skeletal muscle tissue at 3 d (Fig. 5C–F). These data suggested that macrophages within skeletal muscle tissue were efficaciously eliminated by LC injection. The pathological results have indicated that the advantageous influence of ESC–MSC-EVs on skeletal muscle injury induced by ACS was mitigated by the depletion of macrophages (Fig. 5G), and the proportion of injured myofibers increased to 46.06 ± 3.72% (Fig. 5H). The results obtained through Western blotting were in accordance with the pathological findings (Fig. 5I). The expression levels of the inflammatory protein iNOS and the apoptosis protein Cleaved caspase 3 were observed to be elevated after macrophage depletion (Fig. 5J–K). Serological examinations revealed that the advantageous efficacy of ESC–MSC-EVs on myofibers was attenuated by the clearance of macrophages (Fig. 5L). Furthermore, the mRNA expression of inflammatory cytokines demonstrated that the inflammatory response was further augmented in skeletal muscle tissue (Fig. 5M–O). The results indicated that the positive impacts of ESC–MSC-EVs mainly depended on their interactions with macrophages.

### ESC–MSCs-EVs were engulfed by macrophages and induced a transformation of M1 macrophages into M2 phenotype in vitro

**Fig. 6:**
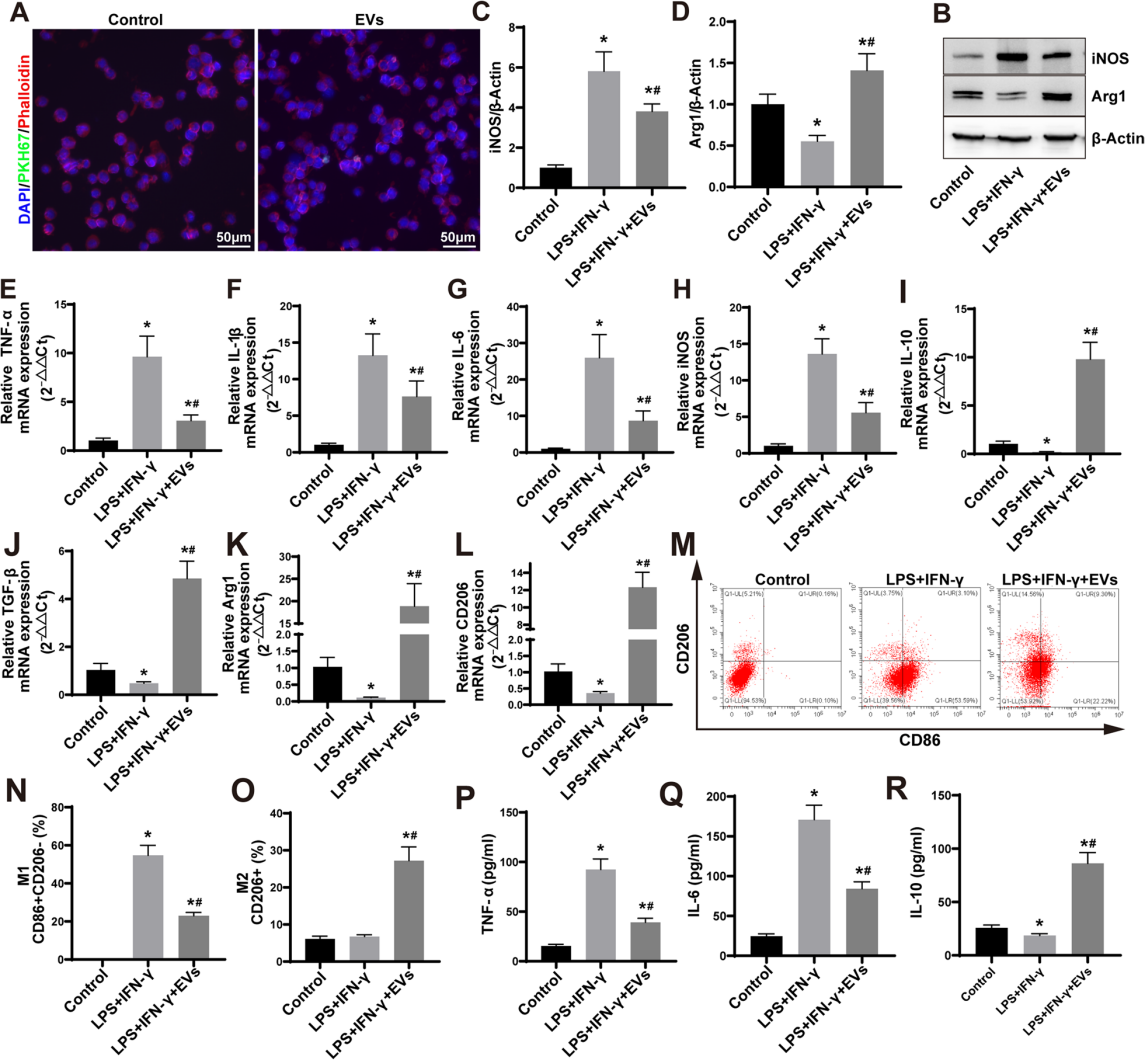
The impact of ESC–MSC-EVs on macrophage polarization within an inflammatory environment. **A** Representative images showcasing the internalization of PKH67-labeled EVs (green) by THP-1 macrophages (DAPI blue), along with fluorescence uptake comparisons involving control and dye-only samples. Scale bar: 50 μm. **B** Representative images of iNOS and Arg1 expression levels of THP-1 macrophages by Western blotting at 48 h. **C**–**D** Quantitative analysis expression of iNOS and Arg1 (*n* = 6). **E**–**H** The gene expression of TNF-α, IL-1β, IL-6, and iNOS in THP-1 macrophages at 48 h was measured via RT-qPCR (*n* = 6). **I**–**L** The gene expression of IL-10, TGF-β, Arg1, and CD206 in THP-1 macrophages at 48 h was measured via RT-qPCR (*n* = 6). **M** Illustrative flow cytometry plots demonstrating the proportions of M1 (CD86 + CD206-) and M2 (CD206 +) phenotypes after 48 h. **N**–**O** Quantification of M1 and M2 macrophage proportion obtained through flow cytometry analysis. **P**–**R** The concentration of cytokines TNF-α, IL-6, and IL-10 in the supernatants of THP-1 cells at 48 h (*n* = 6). Data are presented as the mean ± SD. *P < *0.05* versus control group and ^***#***^*P* < *0.05* versus LPS + IFN-γ group. The blots of Fig. 6B were all cropped, and full-length blots were presented in Additional file 2: Fig. S6

To gain a deeper comprehension of the interactions between ESC–MSC-EVs and macrophages, ESC–MSC-EVs were added to the cultured THP-1 cell. The PKH67-labeled ESC–MSC-EVs were internalized by the macrophages and distributed within the cytoplasm within 6 h (Fig. 6A; Additional file 1: Fig. S4). In order to substantiate the direct influence of ESC–MSC-EVs on macrophages, a culture system was employed wherein LPS and IFN-γ were added prior to the introduction of ESC–MSC-EVs, thereby inducing an inflammatory environment and M1 macrophage polarization, ESC–MSC-EVs were added to the stimulated macrophages. Subsequently, we conducted assessments of macrophage marker protein, mRNA expression levels of inflammatory factors, surface markers, and secretion of inflammatory factors. The Western blotting results demonstrated that ESC–MSC-EVs reduced the expression of the inflammatory protein iNOS in macrophages, while simultaneously increasing the expression of the anti-inflammatory protein Arg1 (Fig. 6B–D). Quantitative real‑time PCR analysis demonstrated a significant downregulation of mRNA expression levels of pro-inflammatory factors such as TNF-α, IL-1β, IL-6, and iNOS in macrophages treated with ESC–MSC-EVs, whereas the expression levels of anti-inflammatory factors IL-10, TGF-β, Arg1, and CD206 were upregulated (Fig. 6E–L). Flow cytometry analysis showed a significant increase in the proportion of M2 macrophages and a considerable decrease in the proportion of M1 macrophages in the group treated with ESC–MSC-EVs (Fig. 6M–O). Furthermore, we analyzed the concentrations of TNF-α, IL-6, and IL-10 in the culture supernatants and observed that ESC–MSC-EVs suppressed the production of inflammation-induced TNF-α and IL-6, while concurrently upregulating the expression of IL-10 (Fig. 6P–R). In summary, those data highlight that ESC–MSC-EVs contribute to the polarization of M2 macrophages while concurrently inhibiting M1 macrophage polarization.

### ESC–MSC-EVs regulate macrophage polarization by affecting NF-κB, JAK-STAT, and PI3K-AKT pathways

**Fig. 7:**
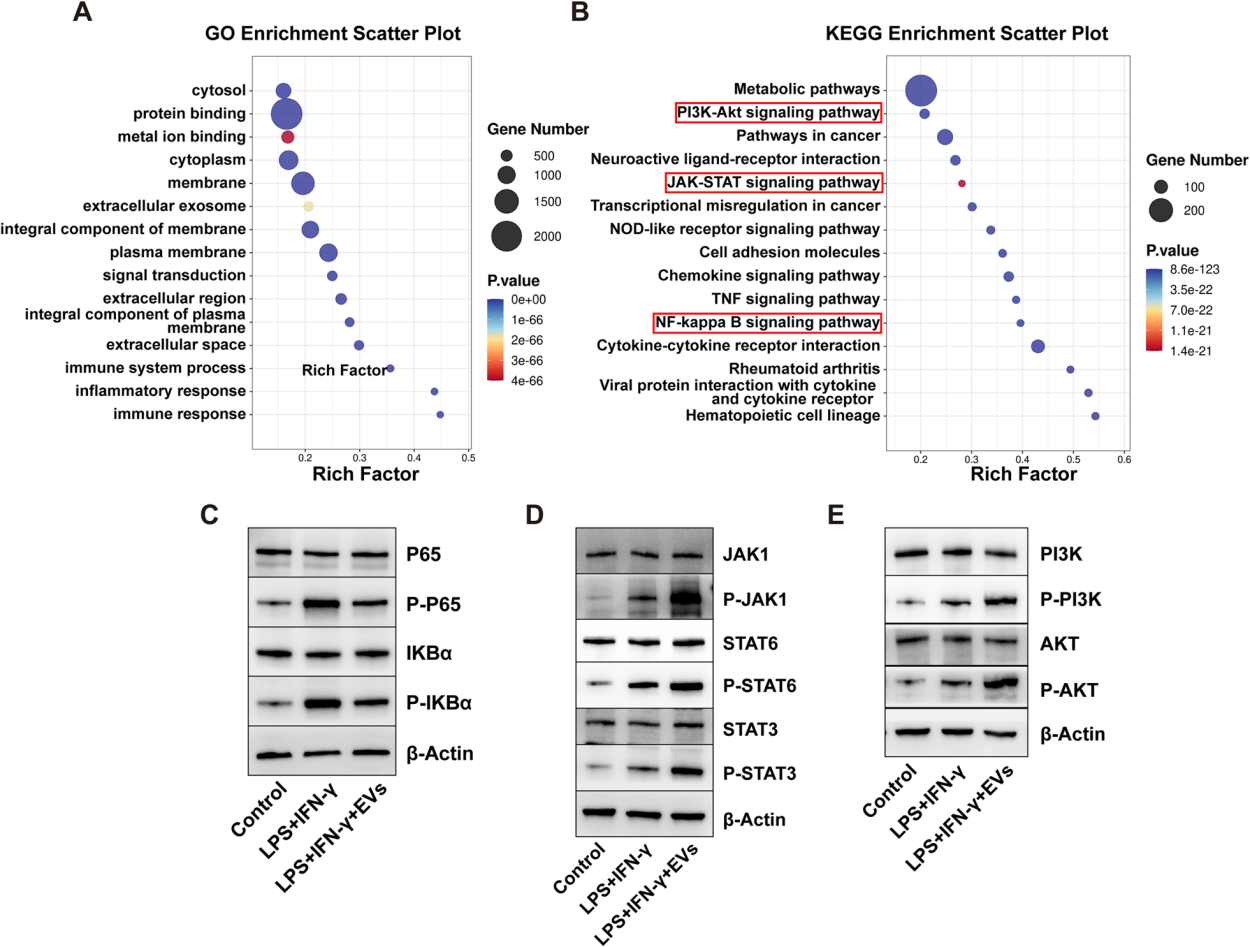
Effects of ESC–MSC-EVs on NF-κB, JAK-STAT, and PI3K-AKT pathways. **A**, **B** The GO and KEGG analysis of the significantly expressed genes between LPS + IFN-γ and LPS + IFN-γ + EVs groups. **C**–**E** Several key NF-κB, JAK-STAT, and PI3K-AKT-related proteins were checked by Western blotting. The blots of Fig. 7C–E were all cropped, and full-length blots were presented in Additional file 2: Figs. S7–S9

In light of the observed ability of ESC–MSC-EVs to regulate macrophage polarization, we undertook transcriptome sequencing analysis to investigate its potential mechanisms. Specifically, we examined the differential expression of genes between the LPS + IFN-γ and LPS + IFN-γ + EVs groups. A total of 3947 genes were identified as being differentially expressed (|log2*FC*|> = 1 and *q* < 0.05). Following treatment with EVs, macrophages exhibited an upregulation of 2200 genes and a downregulation of 1749 genes (Additional file 1: Fig. S5A and B). By utilizing GO bioinformatic analysis, it was determined that the mRNAs showing differential expression are actively involved in complex mechanisms such as signal transduction and protein binding (Additional file 1: Fig. S5C). The statistical analysis of the GO enrichment analysis and the KEGG results was conducted by ggplot2, and the results were visually represented through scatter plots (Fig. 7A and B). The significantly affected KEGG pathways were compared with signaling pathways associated with macrophage polarization, and we observed that the NF-κB, JAK-STAT, and PI3K-AKT pathways were considerably enriched. The involvement of these pathways plays a critical role in macrophage polarization [32, 33]. Consequently, we assessed the key protein levels of prominent NF-κB, JAK-STAT, and PI3K-AKT pathways in macrophages to corroborate our conjecture. Our results demonstrate that ESC–MSC-EVs hinder the activation of the NF-κB pathway while stimulating the activation of the JAK-STAT pathway and PI3K-AKT pathway in inflammatory macrophages (Fig. 7C–E; Additional file 1: Fig. S6A-C). Collectively, these data suggest that ESC–MSC-EVs regulate macrophage polarization by affecting NF-κB, JAK-STAT, and PI3K-AKT pathways.

### miRNAs associated with macrophage polarization were present in ESC–MSC-EVs

**Fig. 8:**
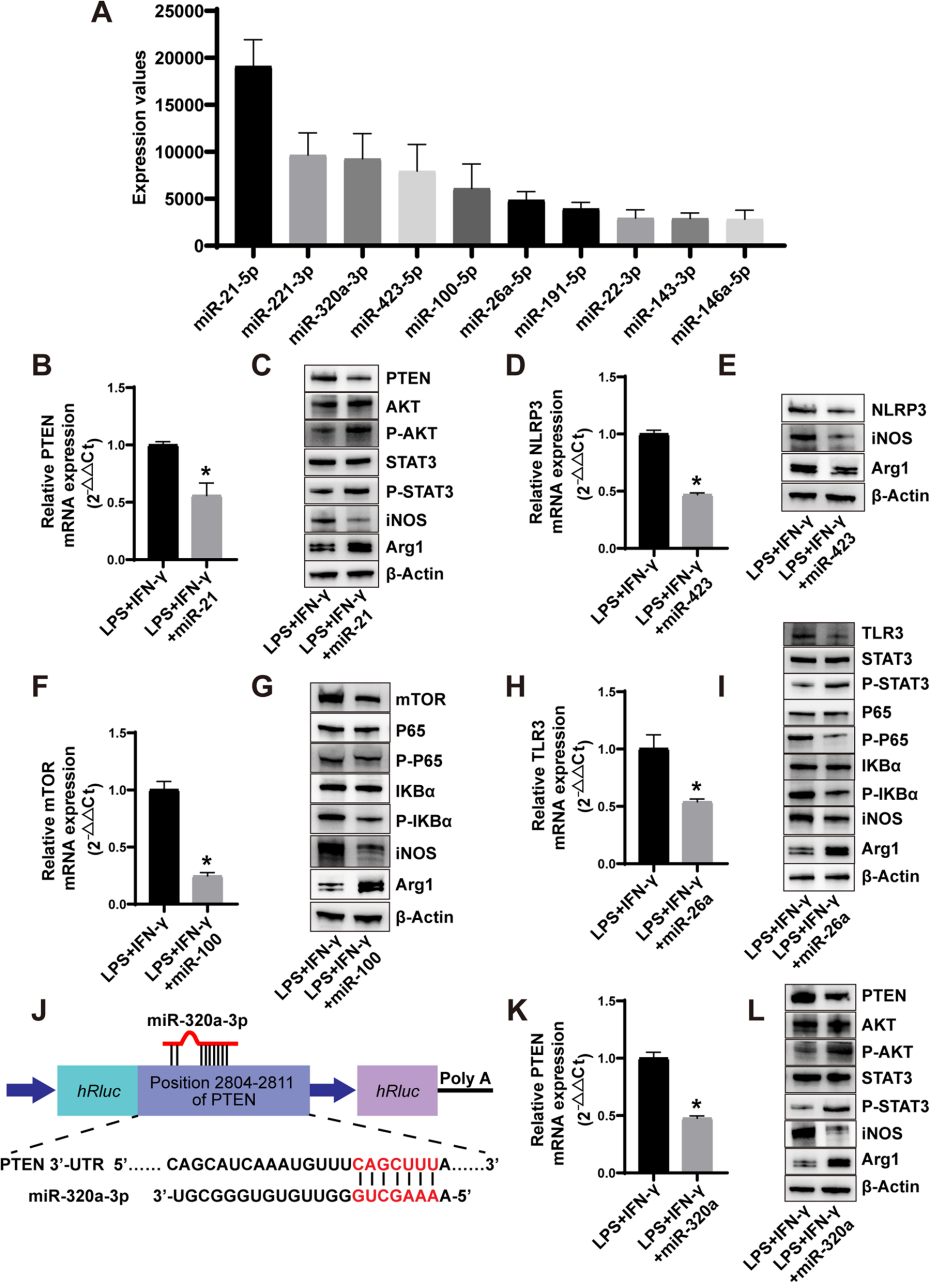
ESC–MSC-EVs-derived miRNAs regulate macrophage polarization by targeting *PTEN*, *NLRP3*, *mTOR*, and *TLR3* mRNAs. **A** The top 10 ranked expression values of miRNAs in ESC–MSC-EVs (*n* = 3). **B**, *PTEN* mRNA level in THP-1 cells transfected with either a mimic NC or a miR-21 mimic (*n* = 3). **C** Representative images of PTEN, AKT, P-AKT, STAT3, P-STAT3, iNOS, and Arg1 expression levels. **D** *NLRP3* mRNA level in THP-1 cells transfected with mimic NC or miR-423 mimic (*n* = 3). **E** Representative images of NLRP3, iNOS, and Arg1 expression levels. **F** *mTOR* mRNA level in THP-1 cells transfected with either a mimic NC or a miR-100 mimic (*n* = 3). **G** Representative images of mTOR, P65, P-P65, IKBα, P-IKBα, iNOS, and Arg1 expression levels. **H** *TLR3* mRNA level in THP-1 cells transfected with either a mimic NC or a miR-26a mimic (*n* = 3). **I** Representative images of TLR3, STAT3, P-STAT3, P65, P-P65, IKBα, P-IKBα, iNOS, and Arg1 expression levels. **J** The binding site between miR-320a-3p and PTEN. **K** *PTEN* mRNA level in THP-1 cells transfected with either a mimic NC or a miR-320a mimic (*n* = 3). **L** Representative images of PTEN, AKT, P-AKT, STAT3, P-STAT3, iNOS, and Arg1 expression levels. Data are presented as the mean ± SD. *P < *0.05* versus LPS + IFN-γ group. The blots of Fig. 8C, E, G, I, and L were all cropped, and full-length blots were presented in Additional file 2: Figs. S10–S14

As EVs primarily manifest their biological influence by delivering bioactive compounds, including miRNAs, we sought to investigate the plausible ingredients of ESC–MSC-EVs for macrophage polarization regulation. This analysis involved high-throughput small RNA sequencing, allowing us to examine and quantify the abundance of different miRNAs present in ESC–MSC-EVs. To confirm the miRNA responsible for regulating macrophage polarization in ESC–MSC-EVs, we examined the top 10 miRNAs present in the EVs, based on the normalized value determined by the sequenced miRNAs (Fig. 8A). Subsequently, we constructed mimics of those miRNAs (Additional file 1: Table S2) and transfected them into macrophages in an inflammatory state, to determine the roles of these miRNAs in macrophage polarization. The results of flow cytometry demonstrated that miR-423 and miR-100 suppressed M1 macrophage polarization, whereas miR-21, miR-320a, and miR-26a enhanced M2 macrophage polarization (Additional file 1: Fig. S7A–C). In order to uncover the molecular mechanisms underlying the impact of these miRNAs on macrophage polarization, we analyzed their potential targets using a combination of TargetScan and Miranda, coupled with a comprehensive literature search (Table 1). Considering that the miR-21/PTEN, miR-423/NLRP3, miR-100/mTOR, and miR-26a/TLR3 axes have been recognized as the signal pathways of macrophage polarization, we transfecting THP-1 cells with each miRNA mimic and observed a corresponding decrease in the gene expression levels of *PTEN* (Fig. 8B), *NLRP3* (Fig. 8D), *mTOR* (Fig. 8F), and *TLR3* (Fig. 8H). The current studies demonstrate that PTEN inhibits the phosphorylation of AKT and STAT3 proteins, which impedes M2 polarization of macrophages [34]. Conversely, NLRP3 promotes M1 macrophage polarization [35], while mTOR inhibits the polarization of M1 macrophages by suppressing the NF-κB pathway [36]. Additionally, TLR3 can induce the transformation of macrophages from M2 to M1 orchestrating the polarization of the NF-κB signaling pathway, while concurrently impeding the phosphorylation of STAT3 [37]. Then, we examined the protein expression of corresponding signal pathways. Our findings demonstrate that miR-21 leads to a reduction in PTEN protein expression, thereby concurrently advancing the phosphorylation of both AKT and STAT3 (Fig. 8C; Additional file 1: Fig. S8A). Additionally, miR-423 reduces the protein expression of NLRP3 (Fig. 8E; Additional file 1: Fig. S8B). Moreover, miR-100 diminishes the expression of mTOR and impedes the activation of the NF-κB pathway (Fig. 8G; Additional file 1: Fig S8C). Furthermore, miR-26a reduces the protein expression of TLR3, inhibits the activation of the NF-κB pathway, and promotes the phosphorylation of STAT3 (Fig. 8I; Additional file 1: Fig. S8D). To determine the mechanism by which miR-320a regulates macrophage polarization, we analyzed its potential targets using TargetScan and Miranda. Among hundreds of predicted mRNA targets, we focused on *PTEN* (Fig. 8J), which may inhibit the phosphorylation of AKT and STAT3 proteins. After transfected with miR-320a, the gene expression level of *PTEN* was significantly decreased in THP-1 cells (Fig. 8K). miR-320a reduced PTEN protein expression while promoting the phosphorylation of AKT and STAT3 (Fig. 8L; Additional file 1: Fig. S8E). This finding suggests that miR-320a enhanced M2 macrophage polarization by targeting *PTEN*.

**Table 1:** miRNA-targeted gene reported by the literature

miRNA	Targeted gene
miR-21-5p	PTEN [34, 38]
miR-320a-3p	None
miR-423-5p	NLRP3 [39, 40]
miR-100-5p	mTOR [36, 41]
miR-26a-5p	TLR3 [37, 42]

## Discussion

**Fig. 9:**
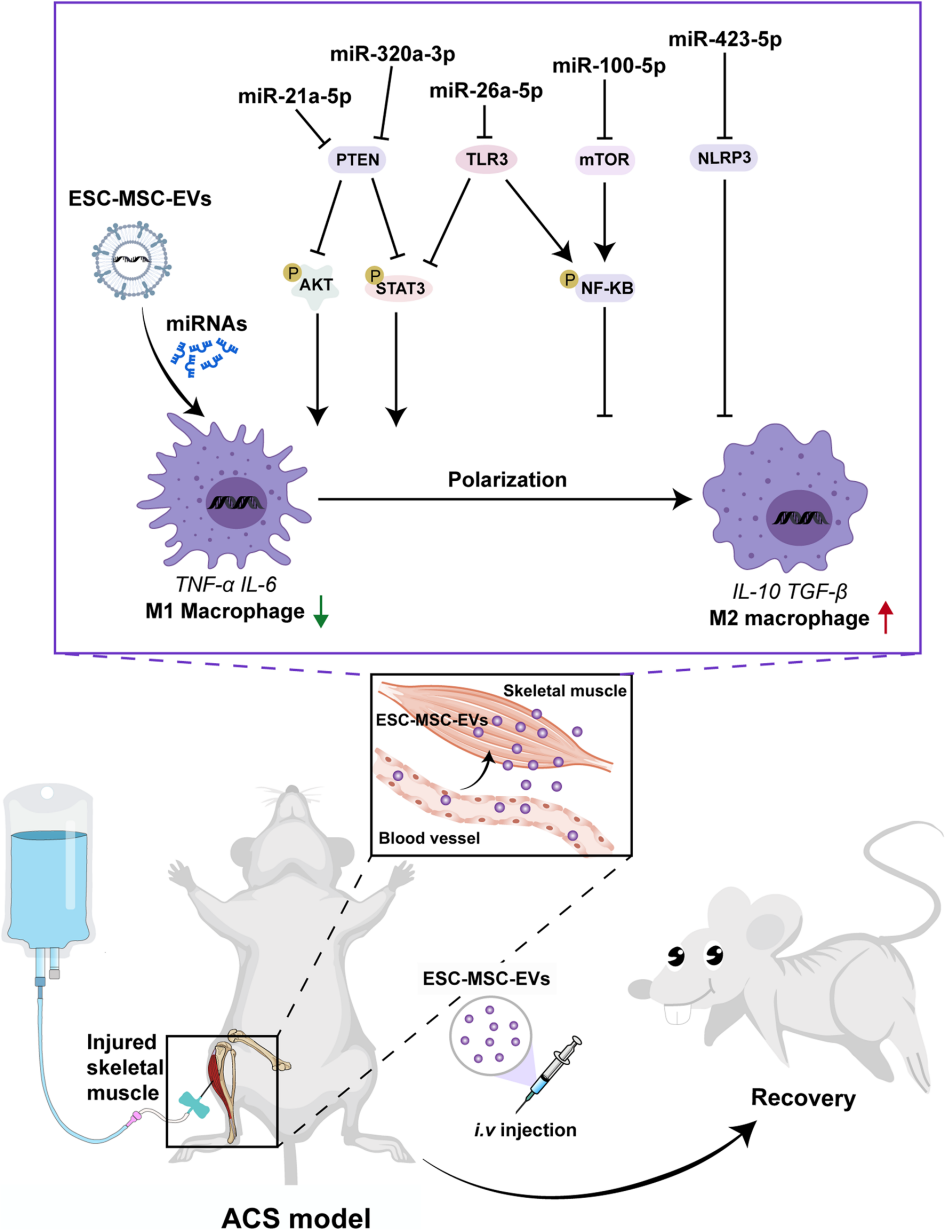
ESC–MSC-EVs ameliorate ACS-induced severe skeletal muscle injury through target macrophage

Our study initially investigated the therapeutic efficacy of ESC–MSC-EVs in the context of ACS-induced severe skeletal muscle injury. Subsequently, we established a direct correlation between the advantageous impacts of ESC–MSC-EVs and their ability to modulate macrophage activity. To validate the significance of macrophages in the ESC–MSC-EVs therapy, we utilized a macrophage depletion model. Moreover, we ascertained that ESC–MSC-EVs effectively reduce the population of pro-inflammatory M1 macrophages while simultaneously promoting the increase in reparative M2 macrophages. This effect is primarily mediated through various miRNAs, including miR-21/PTEN, miR-320a/PTEN, miR-423/NLRP3, miR-100/mTOR, and miR-26a/TLR3 axis (Fig. 9).

After reperfusion following fasciotomy in ACS, an inflammatory cascade is triggered within the muscular tissue due to the release of damage-associated molecules from necrotic myofibers. While the initial pro-inflammatory response clears dead cells and debris, the accumulation of inflammatory cells can cause further damage to surviving skeletal muscle cells [1, 43]. Conventional anti-inflammatory drugs have demonstrated limited efficacy in mitigating the injury, highlighting the imperative for an efficacious therapy that not only diminishes the duration and harm of the inflammatory response but also stimulates reparative pathways [3, 44]. Here, our results suggested that systemic administration of ESC–MSC-EVs improved the skeletal architecture, mitigated myofibers apoptosis, and modulated the expression of molecules associated with apoptosis, consequently reducing systemic inflammation and skeletal muscle damage in the short-term phase of ACS (Fig. 2; Additional file 1: Fig. S2). Additionally, ESC–MSC-EVs therapy stimulated myofibers regeneration during the repair process following skeletal muscle injury and enhanced long-term muscle function recuperation, while also impeding muscle fibrosis (Fig. 3). These data suggest that ESC–MSC-EVs hold significant therapeutic potential in cases of ACS-induced severe skeletal muscle injury. The underlying mechanism is closely tied to their ability to modulate inflammation, which persists not only during the acute inflammatory phase but also throughout the subsequent healing phase.

Macrophages are widely recognized as key players in skeletal muscle inflammation, as they are involved in both the initiation and resolution of the inflammatory process [45]. The current research has illuminated that the intricate interplay between MSCs and macrophages is chiefly reliant on cellular communication via the secretion of EVs [46, 47]. The previous studies have demonstrated the beneficial effect of somatic tissue-derived MSC-EVs on skeletal muscle injury by acting on macrophages [48]. Consequently, we noted the polarization of macrophages in the skeletal muscle tissue following ESC–MSC-EVs therapy, which resulted in a marked reduction in M1 (CD68^+^CD86^+^) infiltration and an increase in M2 (CD68^+^CD206^+^) infiltration within the skeletal muscle tissue. In-depth examination further divulged that the application of ESC–MSC-EVs treatment instigated a discernible diminishment in the expression of pro-inflammatory mediators, while concurrently eliciting an augmentation in the expression of anti-inflammatory agents and neovascularization within the skeletal muscle tissue (Fig. 4; Additional file 1: Fig. S3). After depletion of macrophage, the protective effects of EVs were observed to diminish, indicating that the efficacy of ESC–MSC-EVs treatment is primarily dependent upon their interaction with macrophages (Fig. 5). Our work further confirmed macrophages phagocytosis of ESC–MSC-EVs under inflammatory conditions in vitro, resulting in a transition from M1 to M2 phenotype, concomitant with a decrease in pro-inflammatory factors and an increase in anti-inflammatory repair factors expression (Fig. 6; Additional file 1: Fig. S4). Hence, our findings furnish evidence that the capacity of ESC–MSC-EVs to alleviate the skeletal muscle injury induced by ACS may be attributed to a mechanism that is reliant on macrophages.

To further elucidate the mechanisms by which ESC–MSC-EVs influence macrophage polarization, we performed macrophage transcriptome sequencing comparing LPS + IFN-γ and LPS + IFN-γ + EVs groups. The KEGG pathway analysis unveiled the enriching presence of signaling pathways pertinent to immune regulation, encompassing prominent pathways including NF-κB, JAK-STAT, and PI3K-AKT. Numerous studies have substantiated that the activation of the NF-κB pathway has the potential to induce M1 macrophage polarization. Conversely, the activation of JAK-STAT and PI3K-AKT pathways has been shown to facilitate M2 macrophage polarization [32, 49, 50]. The previous studies on muscle injury have demonstrated that somatic tissue-derived MSC-EVs primarily regulate macrophage polarization by modulating the JAK/STAT pathway [51]. Our data suggested that ESC–MSC-EVs suppressed the phosphorylation of essential proteins in the NF-κB pathway while enhancing the phosphorylation of key proteins in the PI3K-AKT pathway. Additionally, ESC–MSC-EVs promoted the phosphorylation of JAK1, STAT6, and STAT3 proteins in the JAK-STAT pathway (Fig. 7; Additional file 1: Figs. S5–6**)**. These data suggest that NF-κB, JAK-STAT, and PI3K-AKT pathways are essential in the regulation of macrophage status by ESC–MSC-EVs. However, the critical components of ESC–MSC-EVs remain unknown.

The previous studies on muscle injury suggested that somatic tissue-derived MSC-EVs alleviate muscle injury by delivering diverse miRNAs [52, 53]. miRNAs have been identified as crucial constituents of EVs, significantly determining the impact of EVs on target cells. Through their interaction with specific target mRNAs, miRNAs orchestrate the regulation of gene expression at the post-transcriptional level, thereby instigating mRNA degradation or impeding translation progression [54, 55]. Within this investigation, we employed a high-throughput miRNA sequencing approach to scrutinize and quantify the precise expression patterns of miRNAs associated with ESC–MSC-EVs. Our data suggested that ESC–MSC-EVs were rich in a variety of miRNAs regulating macrophage polarization, including miR-21, miR-320a, miR-423, miR-100, and miR-26a. Several axes, including miR-21/PTEN, miR-423/NLRP3, miR-100/mTOR, and miR-26a/TLR3, have been extensively studied for their role in regulating macrophage polarization. Singling out a solitary miRNA might oversimplify the intricate biological landscape of ESC–MSC-EVs, undermining the underlying complexity inherent in their regulatory mechanisms. Our investigations have confirmed the involvement of these axes and demonstrated their synergistic effect in regulating macrophage polarization by ESC–MSC-EVs. Additionally, we have identified the regulatory function of the miR-320a/PTEN axis in macrophage polarization, which has not been reported previously (Fig. 8; Additional file 1: Figs. S7 and S8).

Several limitations should not be ignored in the current study. Firstly, we used the commonly employed method of ultracentrifugation to acquire ESC–MSC-EVs, but achieving a high level of purity for ESC–MSC-EVs is quite challenging. Improved methods are necessary for the future clinical application of ESC–MSC-EVs. Secondly, we are interested in the efficacy and mechanisms of ESC–MSC-EVs in ACS in this study. The distribution of ESC–MSC-EVs within the organs of rats has not been investigated. Therefore, further research in this direction would be conducive to advancing the clinical application of ESC–MSC-EVs. Thirdly, a single dose of ESC–MSC-EVs was chosen to treat the animals in this study, the optimal dosage of ESC–MSC-EVs needs to be confirmed in the future.

## Conclusions

In summary, ESC–MSC-EVs can effectively reduce skeletal muscle injury in ACS rats. ESC–MSC-EVs specifically target macrophages and effectively promote M2 macrophage polarization while simultaneously suppressing M1 macrophage polarization. This modulation of macrophage phenotype is accomplished through the transfer of a diverse range of microRNAs enclosed within the EVs. Consequently, ESC–MSC-EVs are emerging as promising extracellular nanovesicle-based therapeutic modalities for the treatment of ACS (Additional files 3 and 4).

### Supplementary Information

**Additional file 1**. **Fig. S1**: Acquisition and characterization of ESC–MSCs. **Fig. S2**: Representative images (200x) of TUNEL staining of TA muscle sections at 3 d after ACS. **Fig. S3**: Representative images (200x) of immunofluorescence staining for CD206, CD86, and CD31 on muscle tissue sections. **Fig. S4**: Representative images of the uptake of PKH67-labeled EVs (green) by THP-1 macrophages (DAPI blue) and fluorescence uptake with control and dye-only samples. **Fig. S5**: The differential mRNA expression and pathway enrichment analysis of THP-1 cells from the LPS+IFN-γ (A) and LPS+IFN-γ+EVs (B) groups. **Fig. S6**: Quantitative analysis expression of key NF-κB, JAK-STAT, and PI3K-AKT-related proteins expression. **Fig. S7**: Effects of miRNAs on macrophage polarization under inflammatory environment. **Fig. S8**: Quantitative analysis of protein expression in Figure 8. **Table S1**: Sequences for siRNA. **Table S2**: The sequences of the top 10 miRNA.

**Additional file 2**. Corresponding full-length blots.

**Additional file 3**. Transcriptome sequencing data.

**Additional file 4**. Small RNA sequencing data.

## Data Availability

The transcriptome sequencing data have been deposited in the NCBI Sequence Read Archive (SRA) database (https://www.ncbi.nlm.nih.gov/sra/) with the accession number PRJNA1019733. The small RNA sequencing data have been deposited in the NCBI SRA database with the accession number PRJNA1019767. The manuscript and supplemental materials contain all the data collected during the current study, and the corresponding author can provide unprocessed data upon reasonable request.

## Abbreviations

ACS: Acute compartment syndrome
IR: Ischemia–reperfusion
ESC–MSCs: Embryonic stem cells-derived mesenchymal stem cells
ESCs: Embryonic stem cells
EVs: Extracellular vesicles
TA: Tibialis anterior
TEM: Transmission electron microscopy
TUNEL: Terminal dUTP nick end labeling
CK: Creatine kinase
LC: Liposomal clodronate
LV: Liposomal vehicle
