# Supplementary material for: Extracellular vesicles derived from human ESC–MSCs target macrophage and promote anti-inflammation process, angiogenesis, and functional recovery in ACS-induced severe skeletal muscle injury
Source: Stem Cell Res Ther. 2023 Nov 14;14:331. doi: 10.1186/s13287-023-03530-1 (PMC10647154; doi:10.1186/s13287-023-03530-1)
Supplement: Supplementary file 1 — Additional file 1. Fig. S1: Acquisition and characterization of ESC–MSCs. Fig. S2: Representative images (200x) of TUNEL staining of TA muscle sections at 3 d after ACS. Fig. S3: Representative images (200x) of immunofluorescence staining for CD206, CD86, and CD31 on muscle tissue sections. Fig. S4: Representative images of the uptake of PKH67-labeled EVs (green) by THP-1 macrophages (DAPI blue) and fluorescence uptake with control and dye-only samples. Fig. S5: The differential mRNA expression and pathway enrichment analysis of THP-1 cells from the LPS+IFN-γ (A) and LPS+IFN-γ+EVs (B) groups. Fig. S6: Quantitative analysis expression of key NF-κB, JAK-STAT, and PI3K-AKT-related proteins expression. Fig. S7: Effects of miRNAs on macrophage polarization under inflammatory environment. Fig. S8: Quantitative analysis of protein expression in Figure 8. Table S1: Sequences for siRNA. Table S2: The sequences of the top 10 miRNA. [file 13287_2023_3530_MOESM1_ESM.docx]

**Additional file 1**

**Supplemental Figure:**

Supplemental Figure 1. Acquisition and characterization of ESC-MSCs.

Supplemental Figure 2. Representative images (200x) of TUNEL staining of TA muscle sections at 3 d after ACS.

Supplemental Figure 3. Representative images (200x) of immunofluorescence staining for CD206, CD86, and CD31 on muscle tissue sections.

Supplemental Figure 4. Representative images of the uptake of PKH67-labelled EVs (green) by THP-1 macrophages (DAPI blue) and fluorescence uptake with control and dye-only samples.

Supplemental Figure 5. The differential mRNA expression and pathway enrichment analysis of THP-1 cells from the LPS+IFN-γ (A) and LPS+IFN-γ+EVs (B) groups.

Supplemental Figure 6. Quantitative analysis expression of key NF-κB, JAK-STAT, and PI3K-AKT related proteins expression.

Supplemental Figure 7. Effects of miRNAs on macrophage polarization under inflammatory environment.

Supplemental Figure 8. Quantitative analysis of protein expression in Figure 8.

**Supplemental Table:**

Supplemental Table 1. Sequences for siRNA

Supplemental Table 2. The sequences of the top 10 miRNA

**Supplemental Figures**


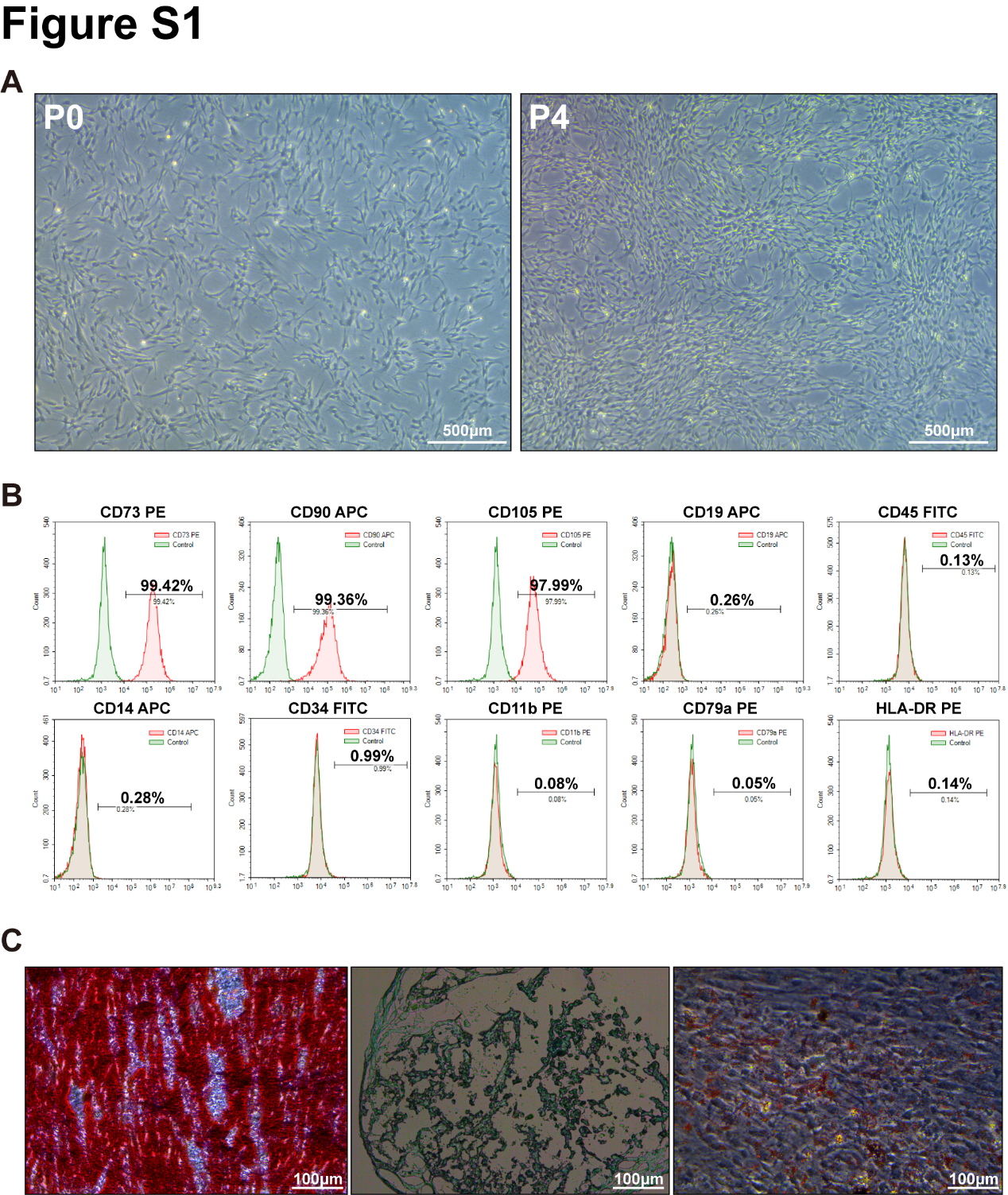


**Supplemental Figure 1. Acquisition and characterization of ESC-MSCs.**

**A**. Representative images of ESC-MSCs at different generations. Scale bar: 500μm. **B**. Flow cytometry was used to examine the surface markers specific to MSCs. Positive markers: CD73, CD90, and CD105; Negative markers: CD19, CD45, CD14, CD34, CD11b, CD79a, and HLA-DR. **C**. Representative images of osteogenic, chondrogenic, and adipogenic differentiation. Scale bar: 500μm.


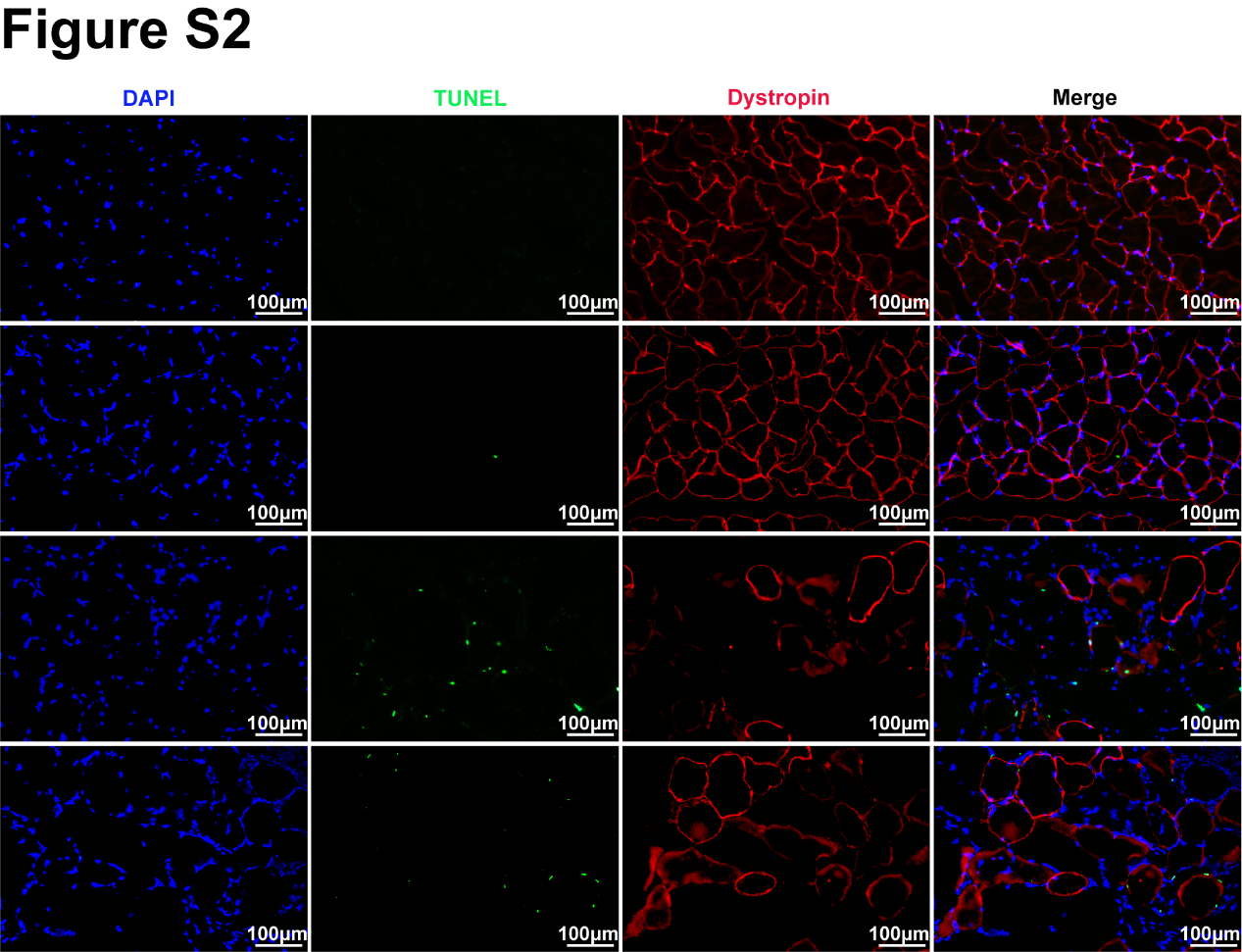


**Supplemental Figure 2. Representative images (200x) of TUNEL staining of TA muscle sections at 3 d after ACS.** Scale bar: 100 μm.


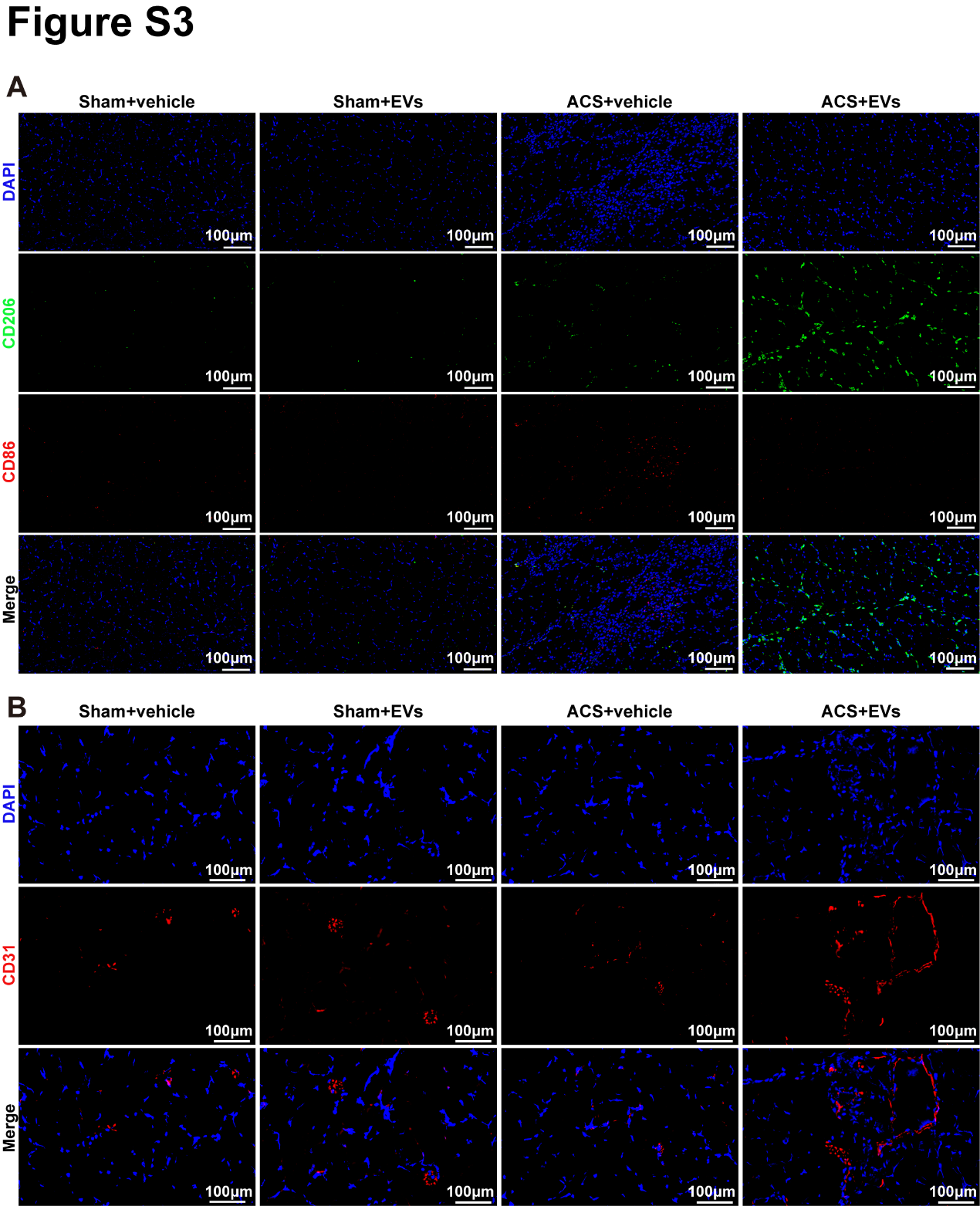


**Supplemental Figure 3. Representative images (200x) of immunofluorescence staining for CD206, CD86, and CD31 on muscle tissue sections.** Scale bar: 100μm.


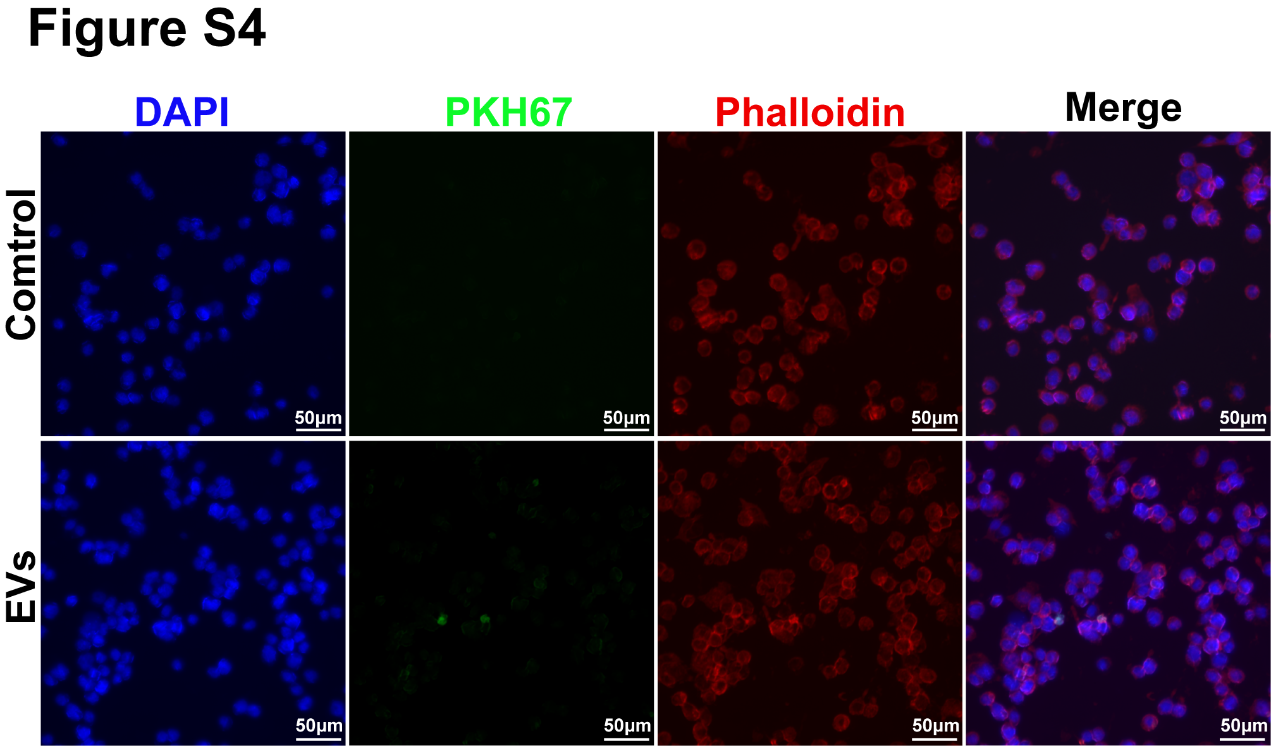


**Supplemental Figure 4. Representative images of the uptake of PKH67-labelled EVs (green) by THP-1 macrophages (DAPI blue) and fluorescence uptake with control and dye-only samples.** Scale bar: 50 μm.


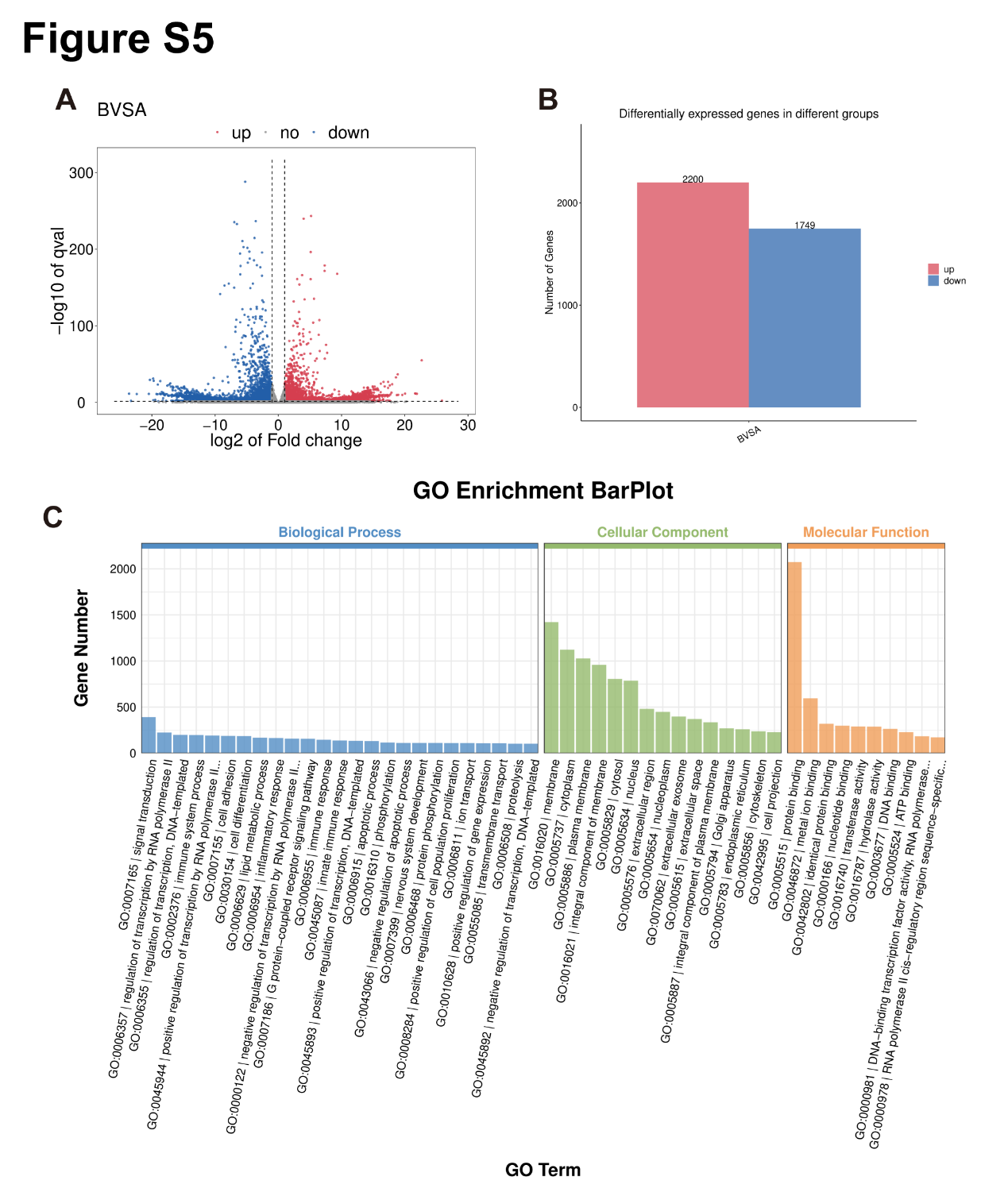


**Supplemental Figure 5. The differential mRNA expression and pathway enrichment analysis of THP-1 cells from the LPS+IFN-γ (A) and LPS+IFN-γ+EVs (B) groups.**

**A**. Volcano plot showing differential gene expression between LPS+IFN-γ and LPS+IFN-γ+EVs groups. **B**. A statistical chart displaying the frequency of down-regulated genes with significantly different expressions is presented below. The red column represents the frequency of up-regulated genes, while the blue column represents the frequency of down-regulated genes. **C**. The GO enrichment analysis of differentially expressed genes revealed the amount of abnormal genes enriched in each entry for functional characterization.


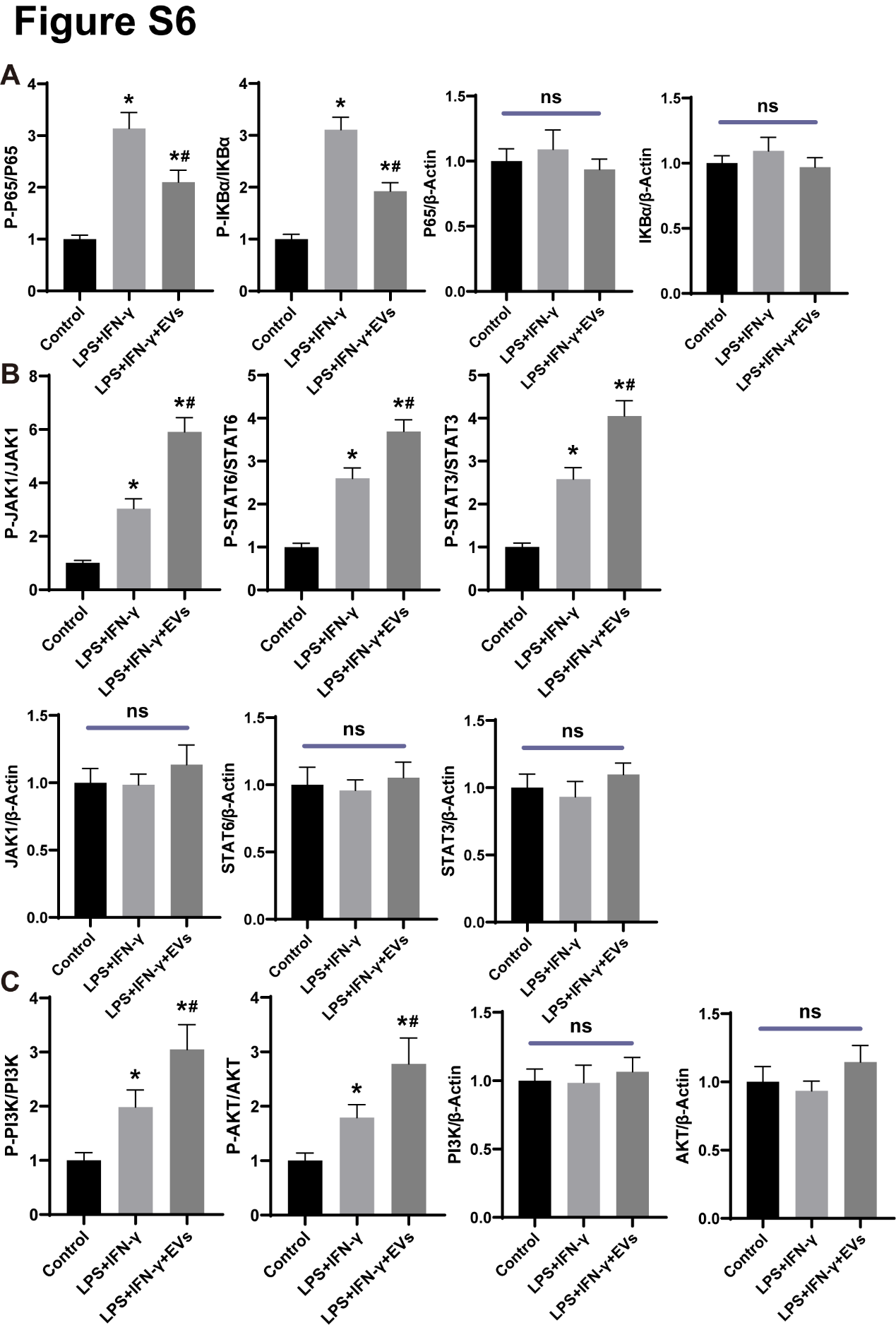


**Supplemental Figure 6. Quantitative analysis expression of key NF-κB, JAK-STAT, and PI3K-AKT related proteins expression.**

**A**. Quantitative analysis the ratio of phosphorylated P65 and IKBα to the corresponding total protein and the expression of P65 and IKBα (n=3). **B**. Quantitative analysis the ratio of phosphorylated JAK1, STAT6, and STAT3 to the corresponding total protein and the expression of JAK1, STAT6, and STAT3 (n=3). **C**. Quantitative analysis the ratio of phosphorylated PI3K and AKT to the corresponding total protein and the expression of PI3K and AKT (n=3). Data are presented as the mean±SD. **P<0.05* versus Control group and ***^#^****P<0.05* versus LPS+IFN-γ group.


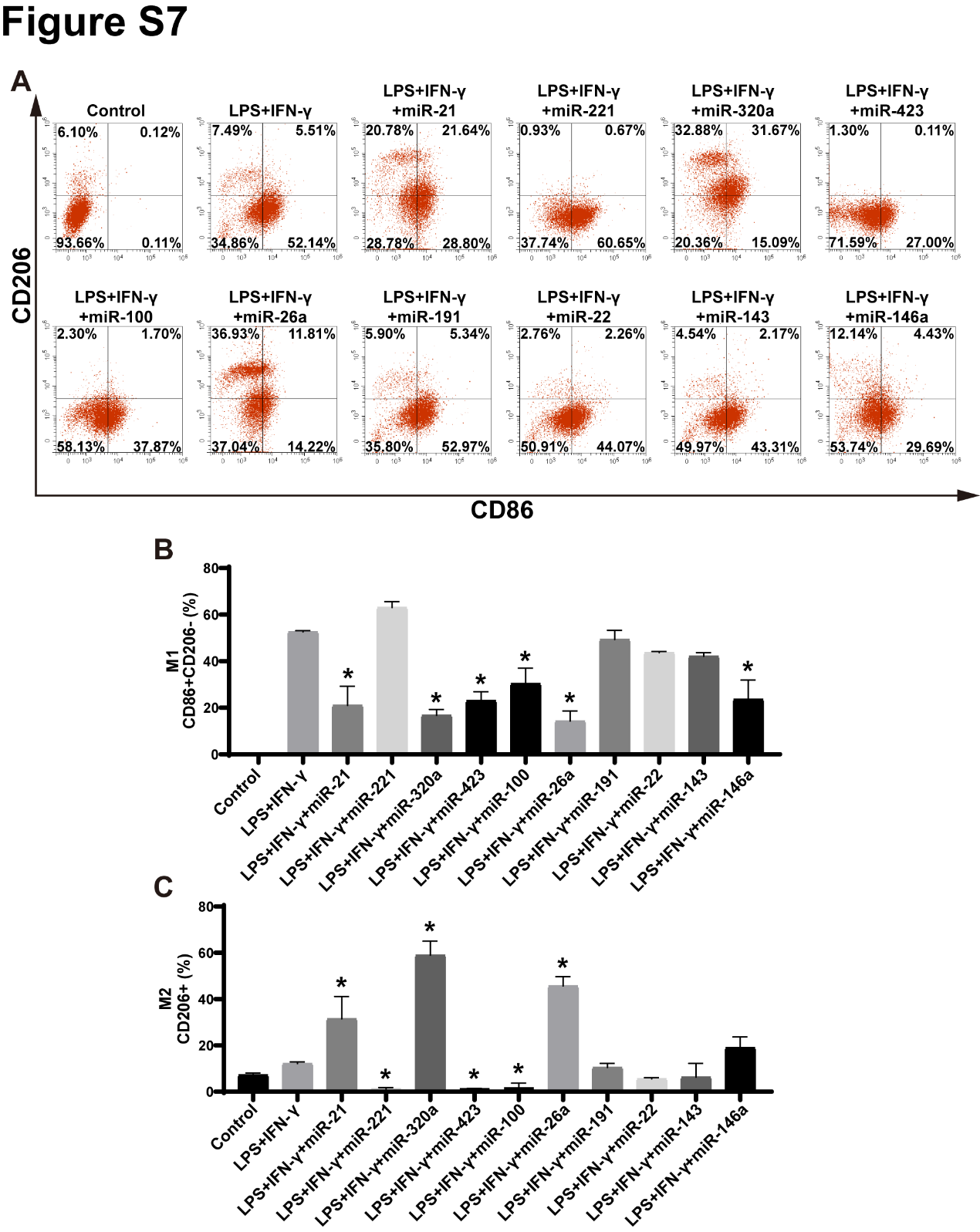


**Supplemental Figure 7. Effects of miRNAs on macrophage polarization under inflammatory environment.**

**A**. Representative flow cytometry plots showing the percentages of M1 (CD86^+^CD206^-^) and M2 (CD206^+^) phenotype at 48 h. **B-C**. Quantification of M1 and M2 macrophage proportion obtained through flow cytometry analysis (n=3). Data are presented as the mean±SD. **P<0.05* versus LPS+IFN-γ group.


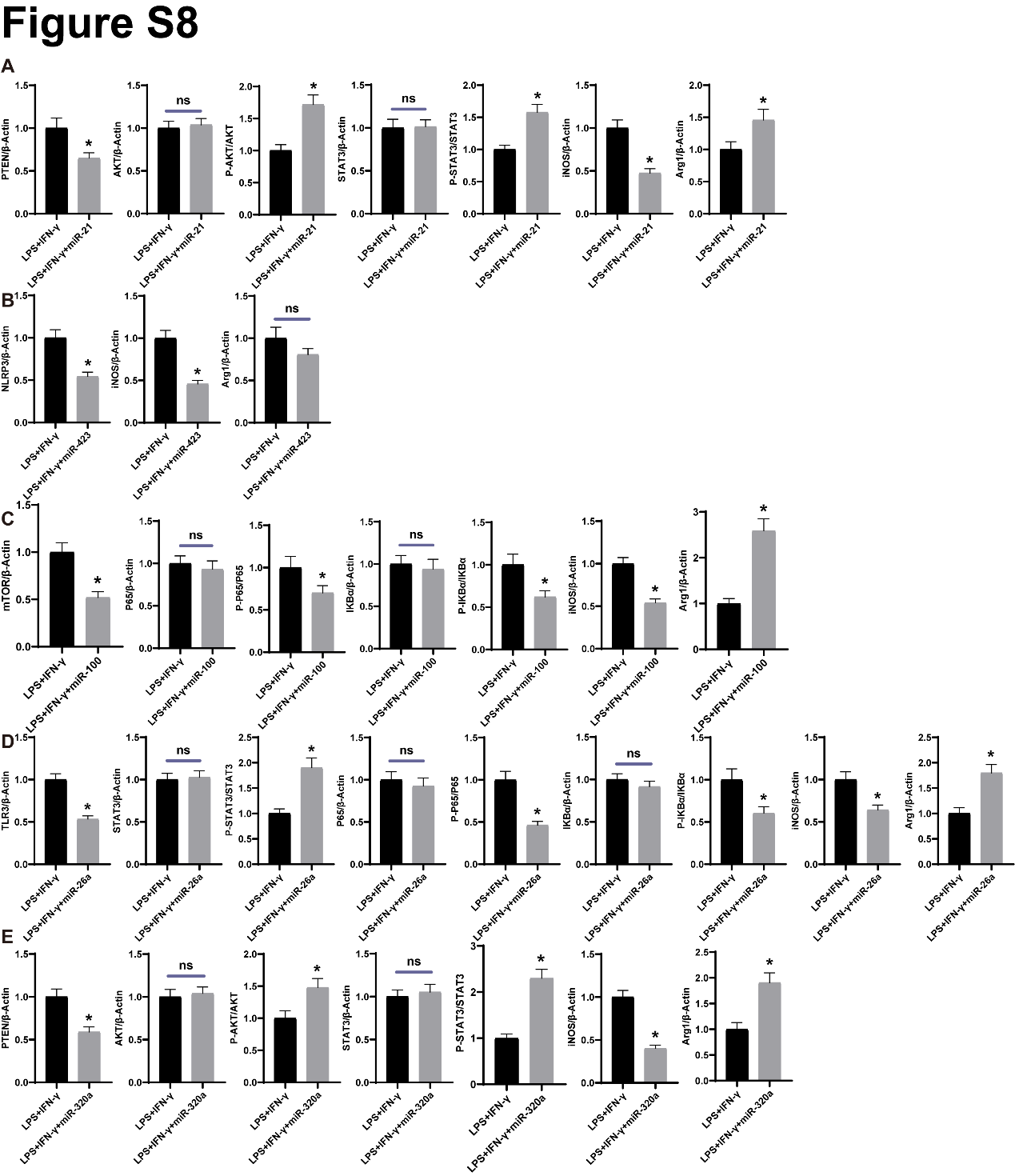


**Supplemental Figure 8. Quantitative analysis of proteins expression in figure8.**

**A**. Quantitative analysis the ratio of phosphorylated AKT and STAT3 to the corresponding total protein and the expression of PTEN, AKT, STAT3, iNOS, and Arg1 (n=3). **B**. Quantitative analysis the expression of NLRP3, iNOS, and Arg1 (n=3). **C**. Quantitative analysis the ratio of phosphorylated P65 and IKBα to the corresponding total protein and the expression of mTOR, P65, IKBα, iNOS, and Arg1 (n=3). **D**. Quantitative analysis the ratio of phosphorylated STAT3, P65 and IKBα to the corresponding total protein and the expression of TLR3, STAT3, P65, IKBα, iNOS, and Arg1 (n=3). **E**. Quantitative analysis the ratio of phosphorylated AKT and STAT3 to the corresponding total protein and the expression of PTEN, AKT, STAT3, iNOS, and Arg1 (n=3). Data are presented as the mean±SD. **P<0.05* versus LPS+IFN-γ group.

**Supplemental Tables**

**Supplemental Table 1. The sequences of primers**

| **Primers** | **Sense/Antisense(5ʼ-3ʼ)** |
| --- | --- |
| Rat-β-Actin-Forward  Rat-β-Actin-Reverse | TGTCACCAACTGGGACGATA GGGGTGTTGAAGGTCTCAAA |
| Rat-TNF-α-Forward  Rat-TNF-α-Reverse | ATGGGCTCCCTCTCATCAGTTCC GCTCCTCCGCTTGGTGGTTTG |
| Rat-IL-6-Forward  Rat-IL-6-Reverse  Rat-IL-10-Forward  Rat-IL-10-Reverse  Rat-VEGFA-Forward  Rat-VEGFA-Reverse  Human-β-Actin-Forward  Human-β-Actin-Reverse  Human-TNF-α-Forward  Human-TNF-α-Reverse  Human-IL-1β-Forward  Human-IL-1β-Reverse  Human-IL-6-Forward  Human-IL-6-Reverse  Human-iNOS-Forward  Human-iNOS-Reverse  Human-IL-10-Forward  Human-IL-10-Reverse  Human-TGF-β-Forward  Human-TGF-β-Reverse  Human-Arg1-Forward  Human-Arg1-Reverse  Human-CD206-Forward  Human-CD206-Reverse  Human-PTEN-Forward  Human-PTEN-Reverse  Human-NLRP3-Forward  Human-NLRP3-Reverse  Human-mTOR-Forward  Human-mTOR-Reverse  Human-TLR3-Forward  Human-TLR3-Reverse | ACTTCCAGCCAGTTGCCTTCTTG TGGTCTGTTGTGGGTGGTATCCTC  CTGCTCTTACTGGCTGGAGTGAAG  TGGGTCTGGCTGACTGGGAAG  GCACTGGACCCTGGCTTTACTG  GGCACACAGGACGGCTTGAAG  CCTGGCACCCAGCACAAT  GGGCCGGACTCGTCATAC  AGCCCTGGTATGAGCCCATCTATC  TCCCAAAGTAGACCTGCCCAGAC  GGACAGGATATGGAGCAACAAGTGG  TCATCTTTCAACACGCAGGACAGG  GACAGCCACTCACCTCTTCAGAAC  GCCTCTTTGCTGCTTTCACACATG  CAGGGTGGAAGCGGTAACAAAGG  CCTGCTTGGTGGCGAAGATGAG  GGGTTGCCAAGCCTTGTCTGAG  CCTTGATGTCTGGGTCTTGGTTCTC  TACAGCAACAATTCCTGGCGATACC  CTCAACCACTGCCGCACAACTC  GGACCTGCCCTTTGCTGACATC  TCTTCTTGACTTCTGCCACCTTGC  TCCGACCCTTCCTTGACTAATCCTC  AGTATGTCTCCGCTTCATGCCATTG  AAACAGTAGAGGAGCCGTCAAATC  TGGATCAGAGTCAGTGGTGTCAG  CCTGGCTGTAACATTCGGAGATTG  TTGCTGCTGAGGACCAAGGAG  CATTCCGACCTTCTGCCTTCAC  CGTTCCTTCTCCTTCTTGACACAG  CAACGACTGATGCTCCGAAGG  TGTTGGCTATGTTGTTGTTGCTTAG |

**Supplemental Table 2. The sequences of the top 10 miRNA**

| **miRNA** | **miRNA sequence (5’-3’)** |
| --- | --- |
| miR-21-5p | UAGCUUAUCAGACUGAUGUUGAC |
| miR-221-3p | AGCUACAUUGUCUGCUGGGUUUC |
| miR-320a-3p  miR-423-5p  miR-100-5p  miR-26a-5p  miR-191-5p  miR-22-3p  miR-143-3p  miR-146a-5p | AAAAGCUGGGUUGUGUGGGCGU  UGAGGGGCAGAGAGCGAGACUUU  AACCCGUAGAUCCGAACUUGUG  UUCAAGUAAUCCAGGAUAGGCU  CAACGGAAUCCCAAAAGCAGCUG  AAGCUGCCAGUUGAAGAACUGU  UGAGAUGAAGCACUGUAGCUC  UGAGAACUGAAUUCCAUGGGUU |
