# Supplementary material for: Extracellular vesicles derived from human ESC–MSCs target macrophage and promote anti-inflammation process, angiogenesis, and functional recovery in ACS-induced severe skeletal muscle injury
Source: Stem Cell Res Ther. 2023 Nov 14;14:331. doi: 10.1186/s13287-023-03530-1 (PMC10647154; doi:10.1186/s13287-023-03530-1)
Supplement: Supplementary file 2 — Additional file 2. Corresponding full-length blots. [file 13287_2023_3530_MOESM2_ESM.docx]

**Additional File 2**

**Corresponding full-length blots.**

**Figure 1 Full-length blots of** **western bloting of Figure 1D.**

Whole membrane

**
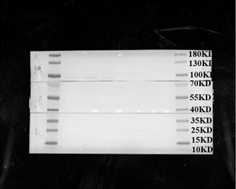
**

Calnexin：90KD


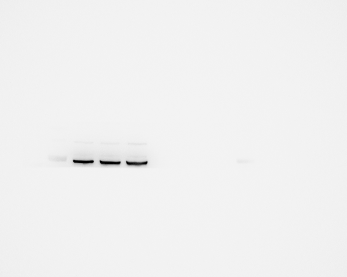

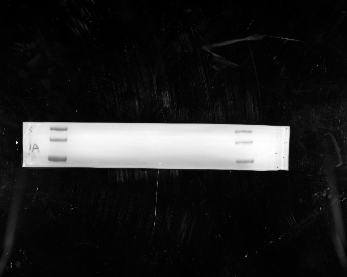

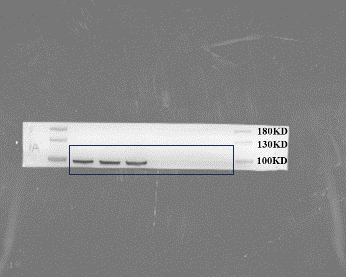


HSP70：70KD


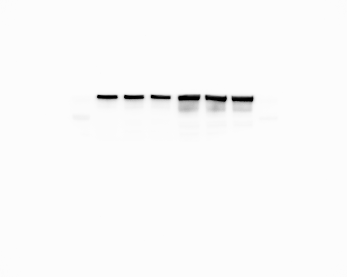

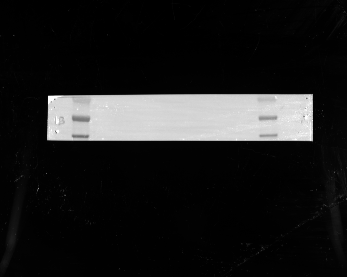

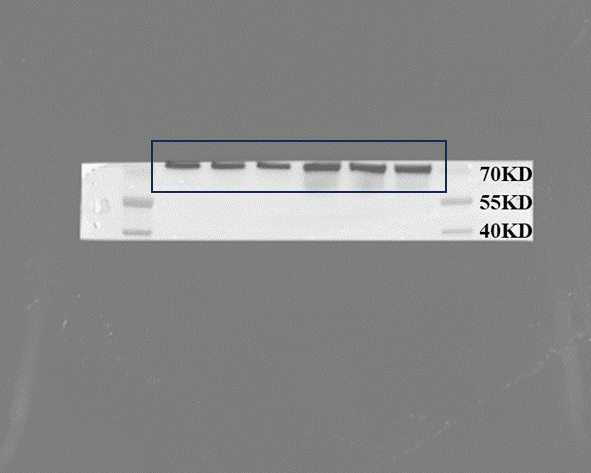


TSG101：47,52KD






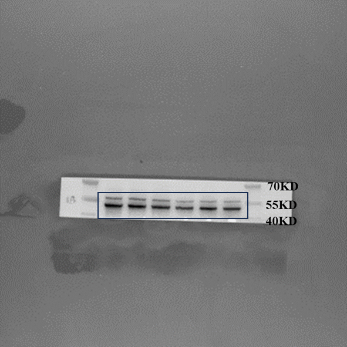


CD63：26KD






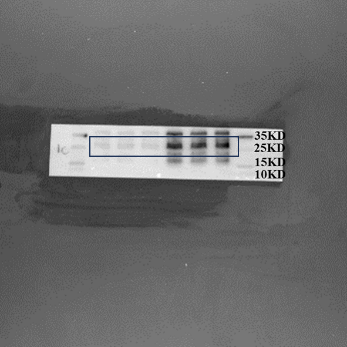


CD9：22-25KD






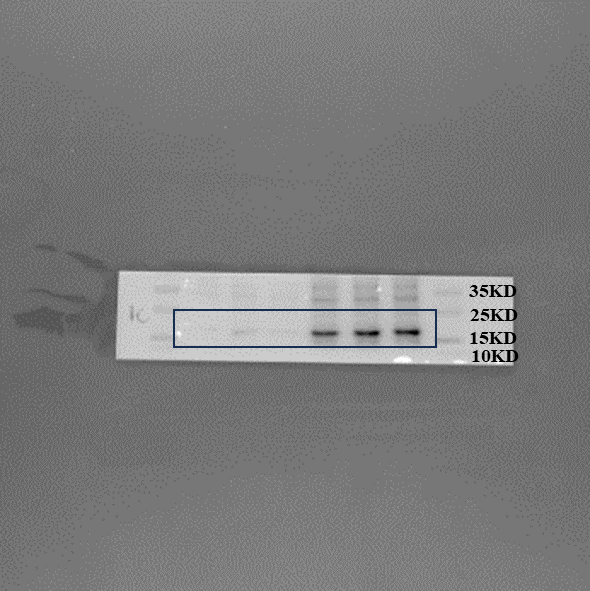


**Figure 2 Full-length blots and original blots generated alternative repeats of western bloting of Figure 2E.**

Whole membrane


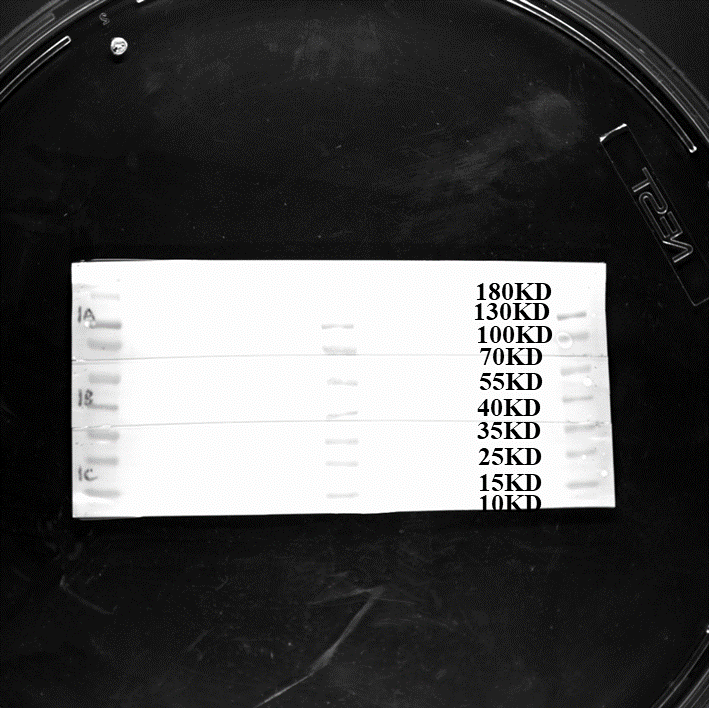

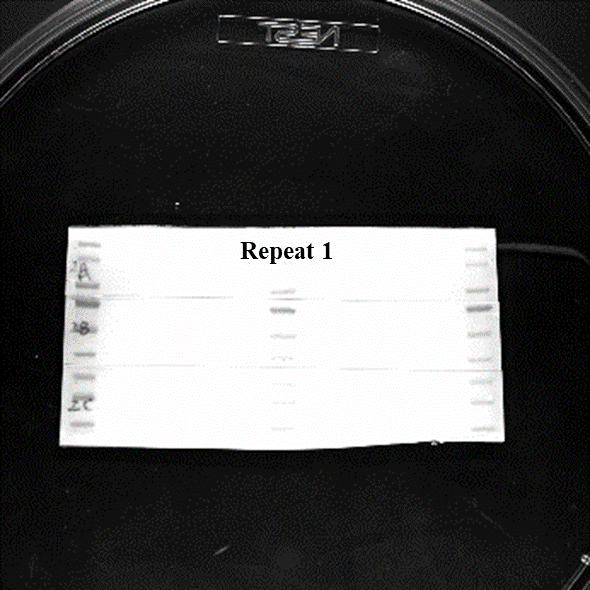

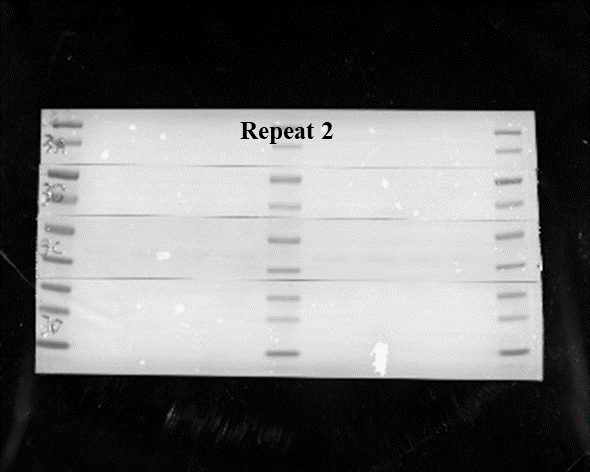


iNOS：130KD


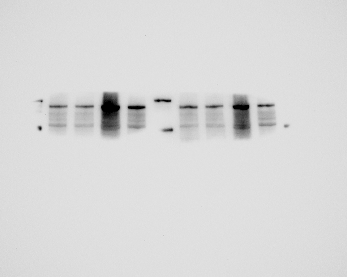

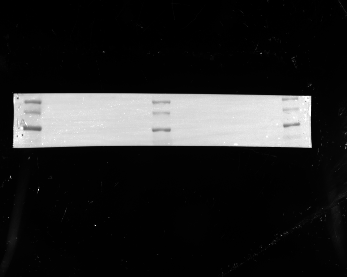

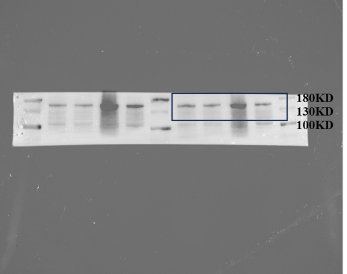






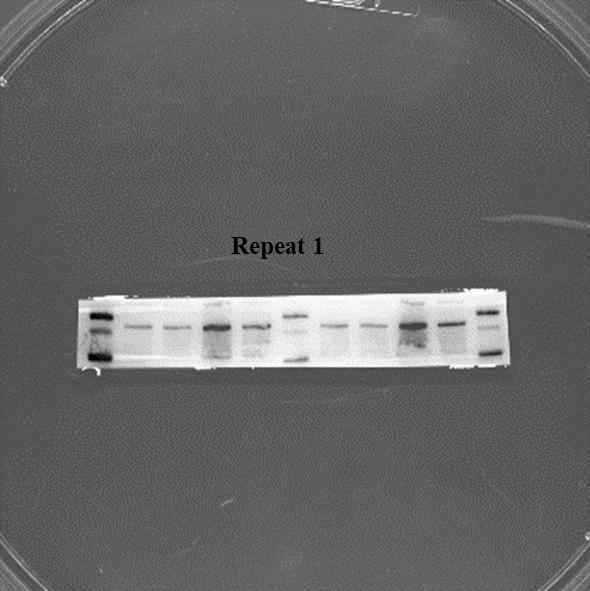


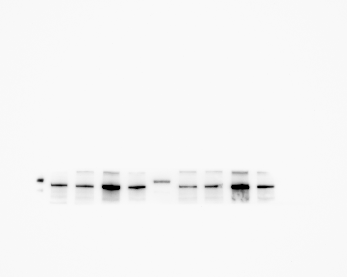

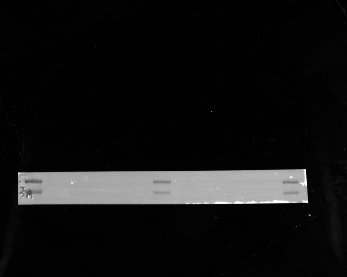

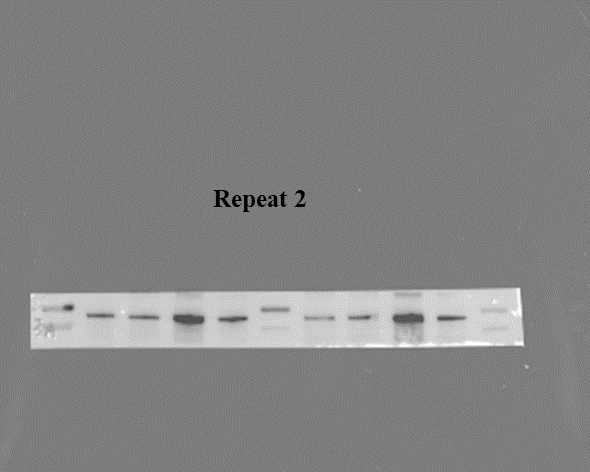


Bcl-2: 26KD






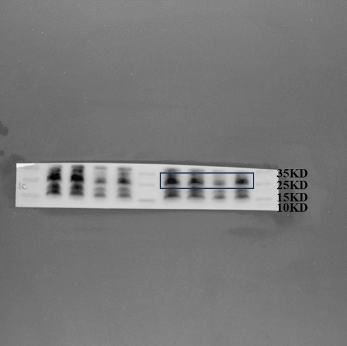






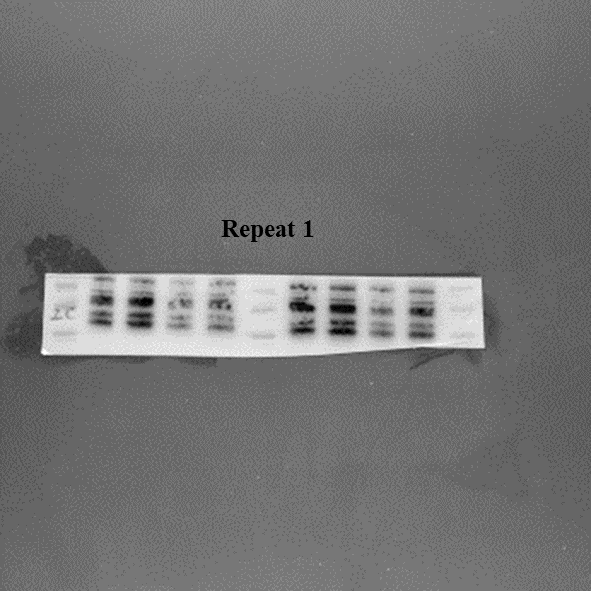


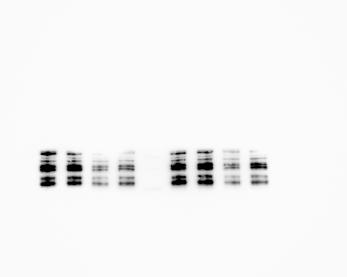

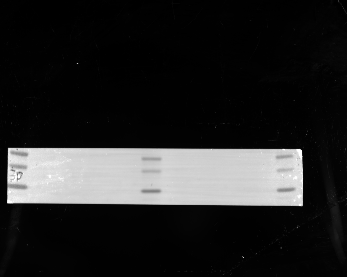

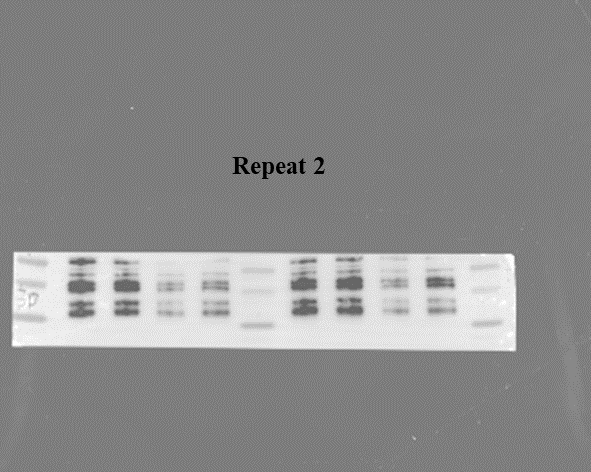


Bax：20KD


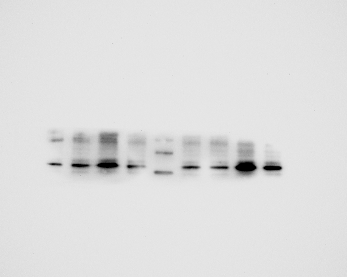

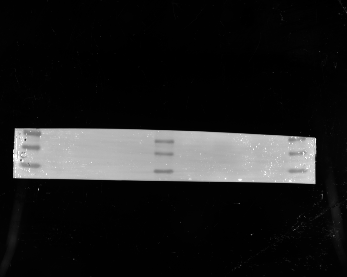

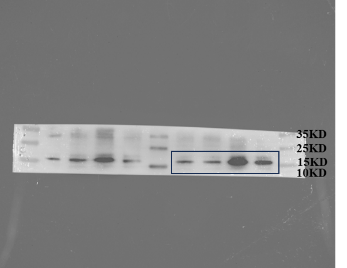






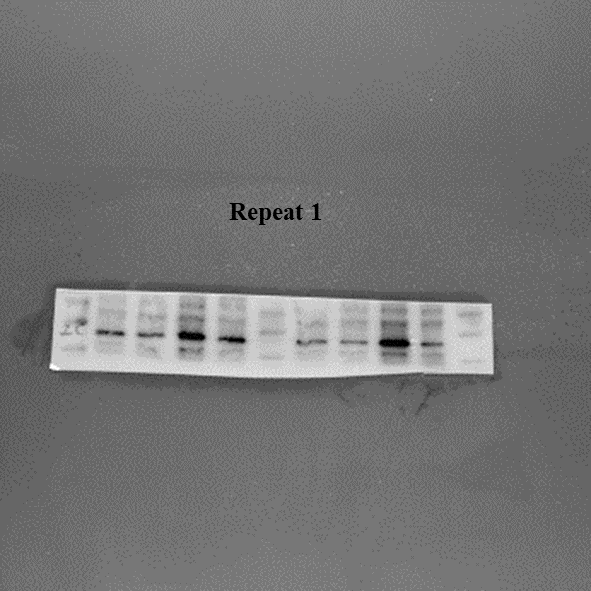


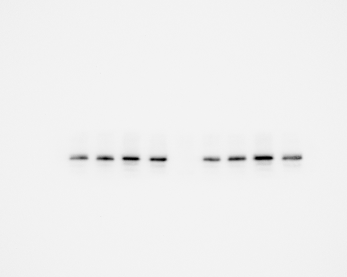

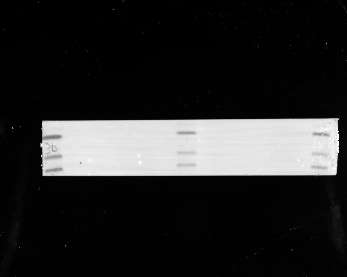

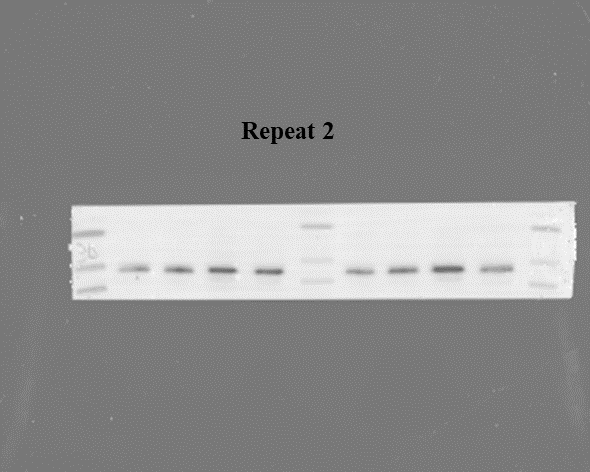


Cleaved caspase3: 17,19KD






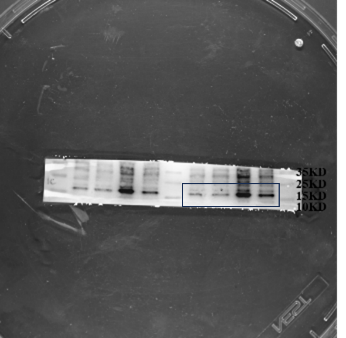


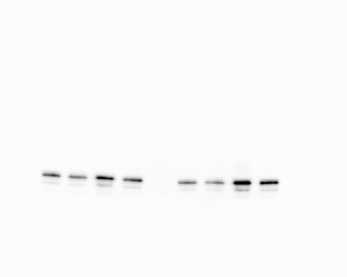

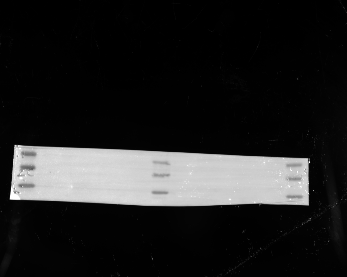

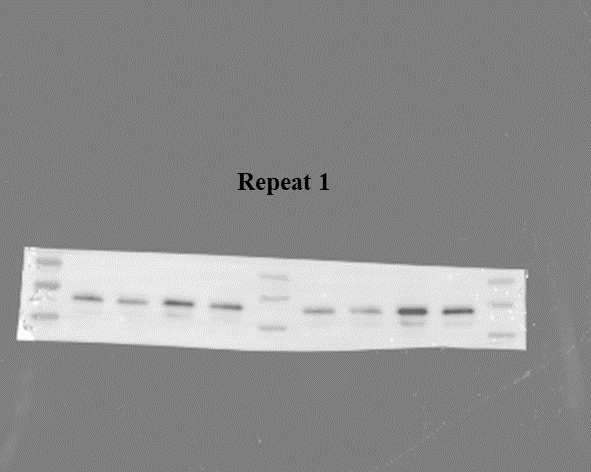


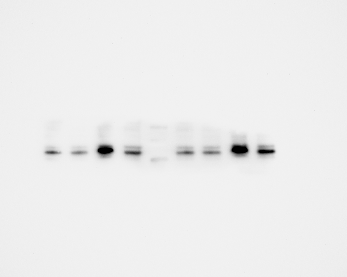

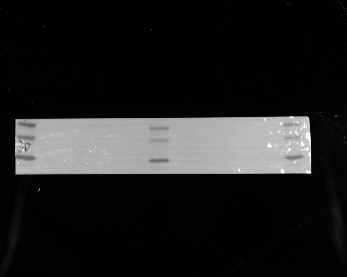

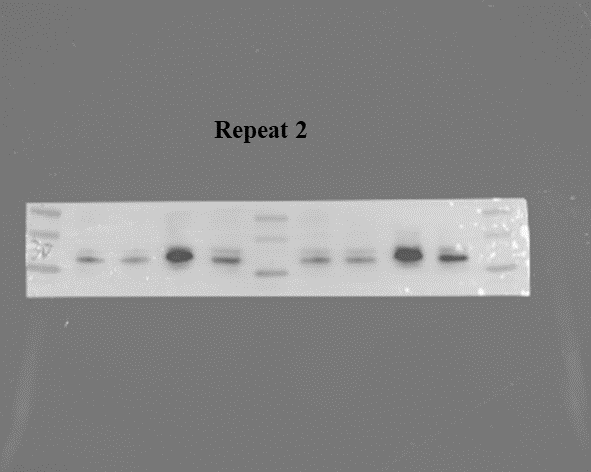


β-Actin: 42KD






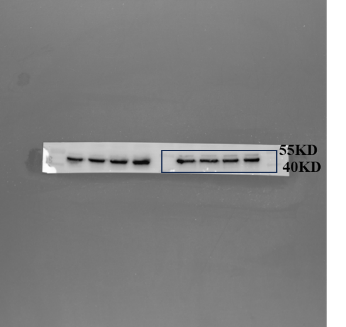






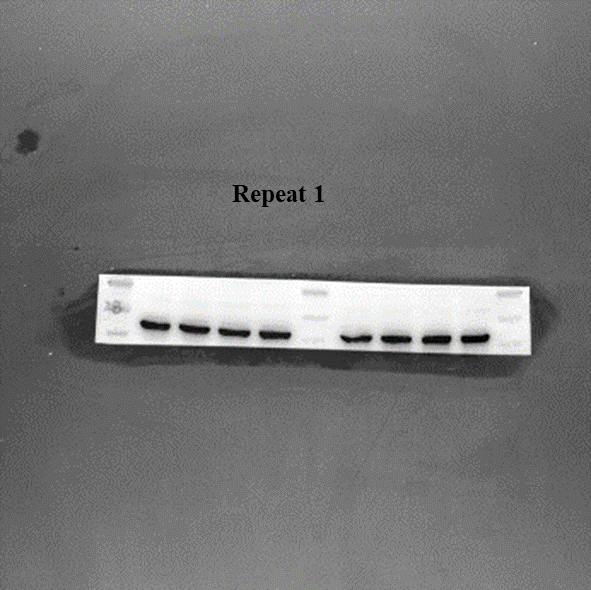


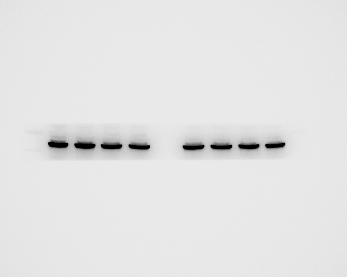

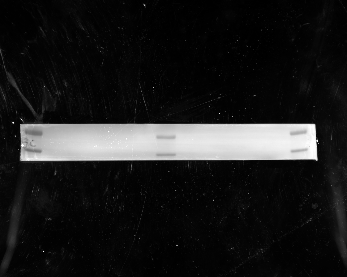

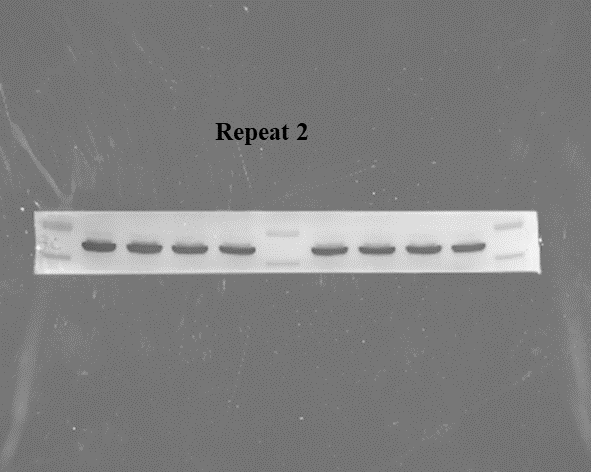


**Figure 3 Full-length blots and original blots generated alternative repeats of western bloting of Figure 4A.**

Whole membrane：


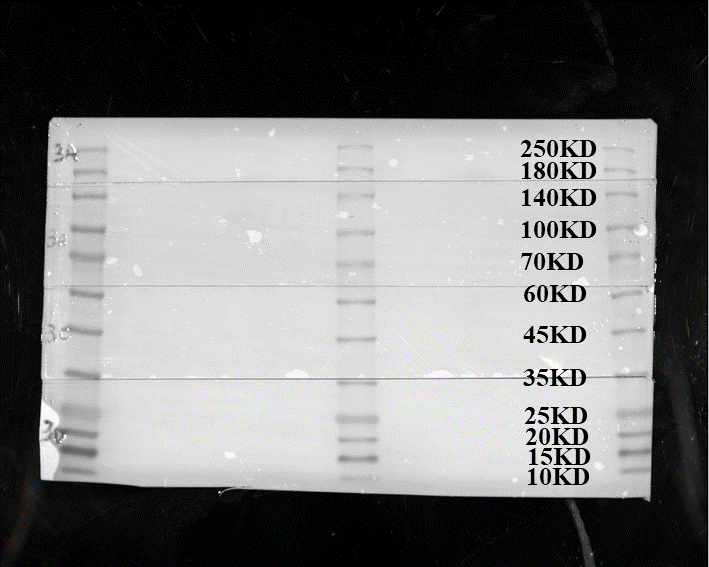

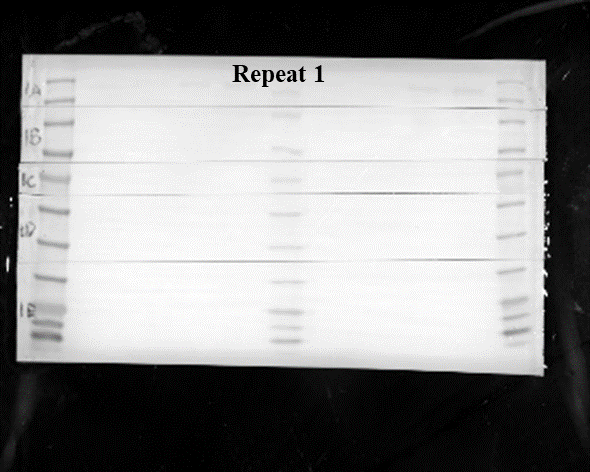

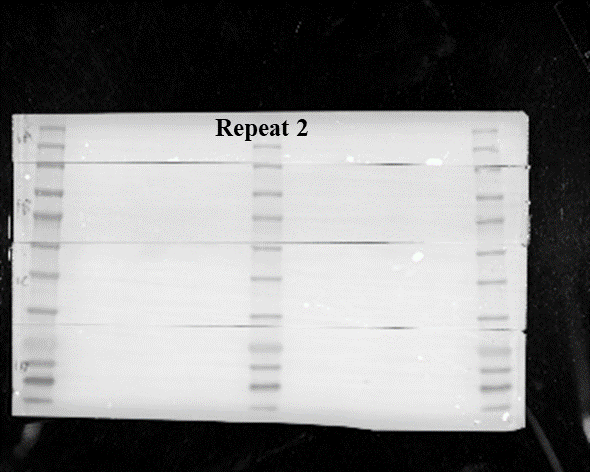


CD206: 245KD


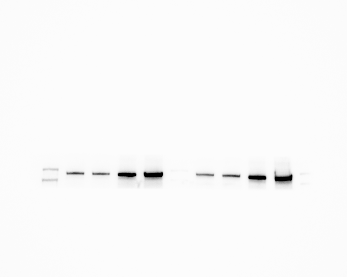

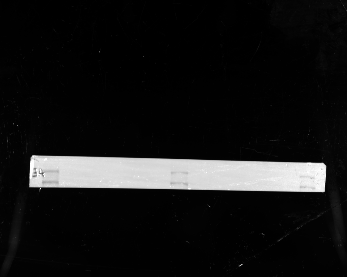

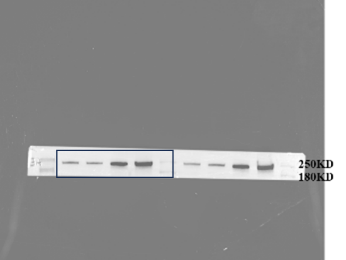


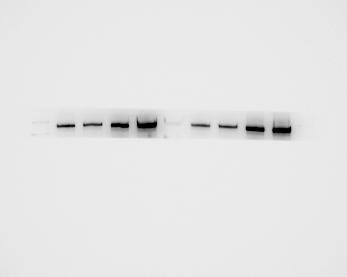

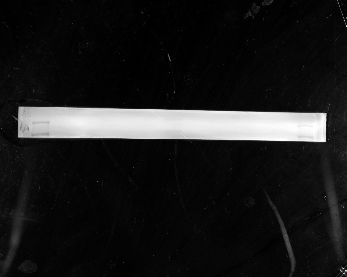

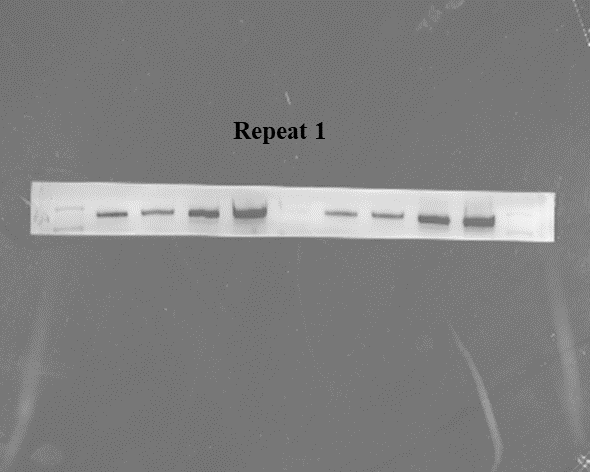


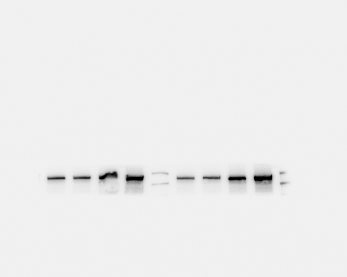

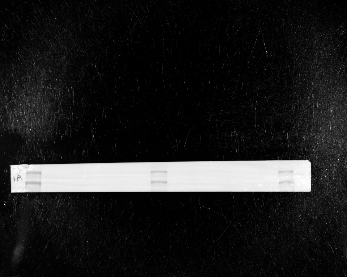

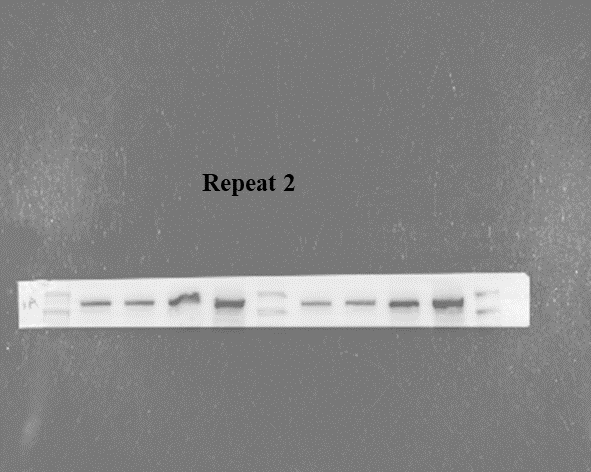


CD68:100KD


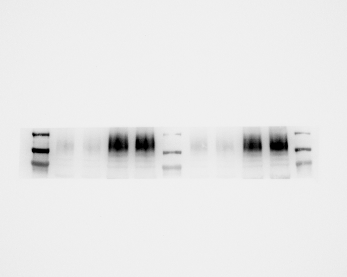

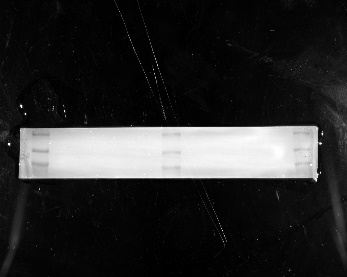

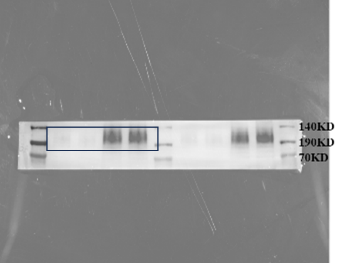


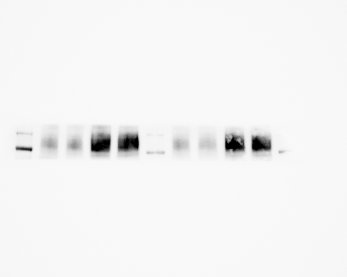

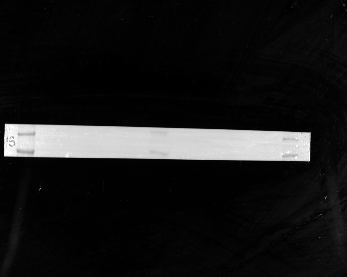

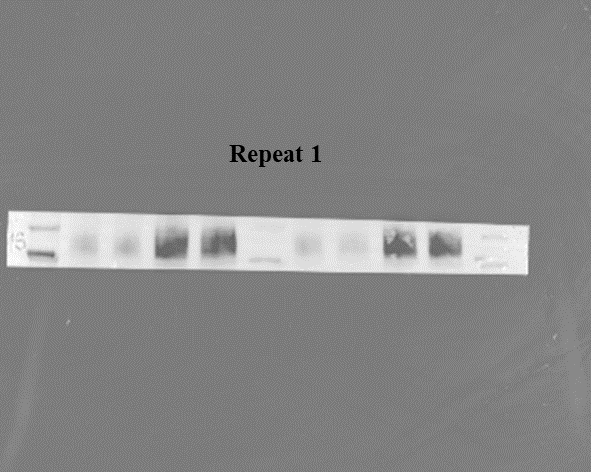


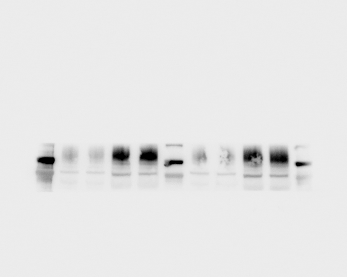

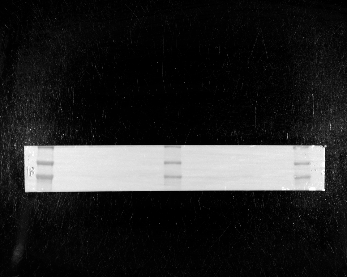

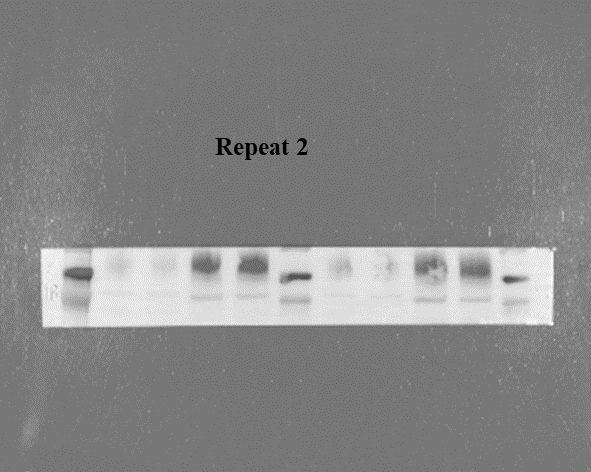


CD86：70KD


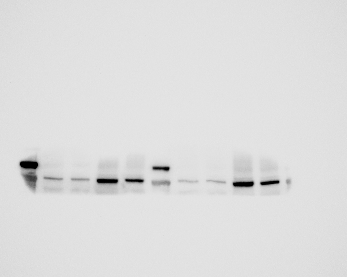

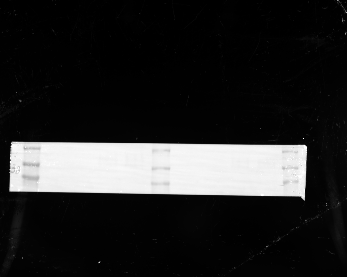

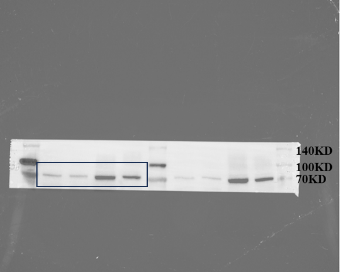


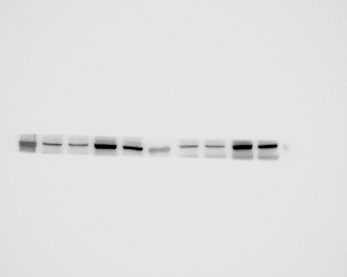

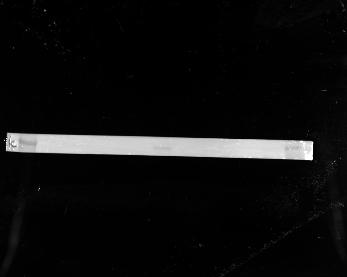

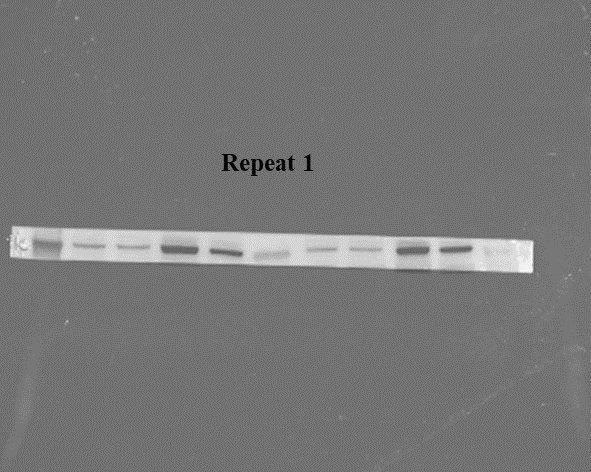


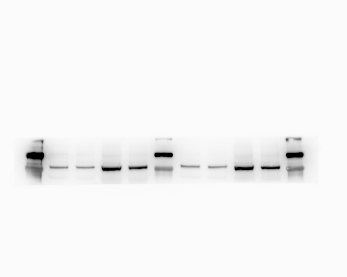

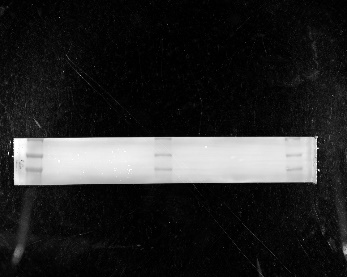

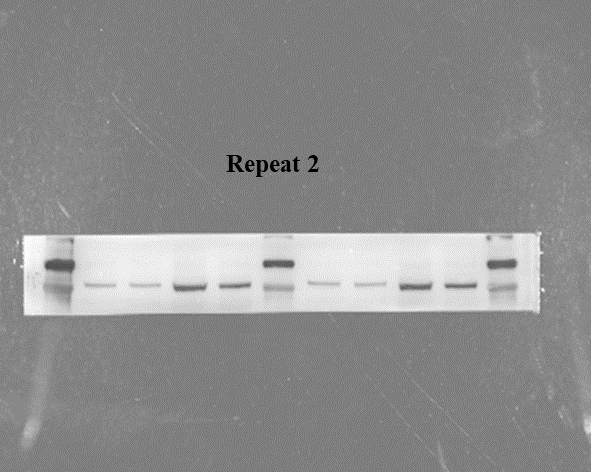


β-Actin: 42KD


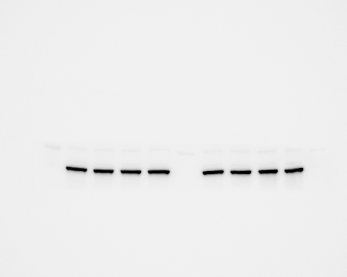

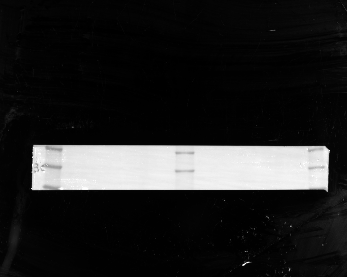

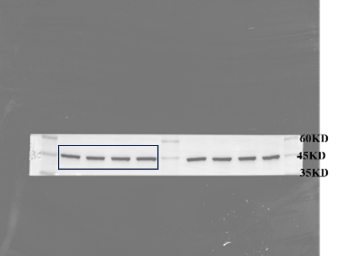


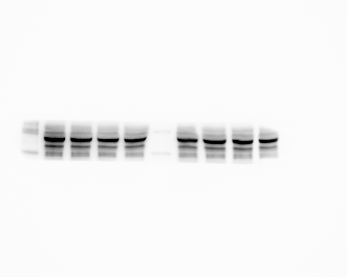

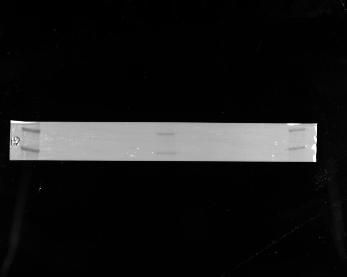

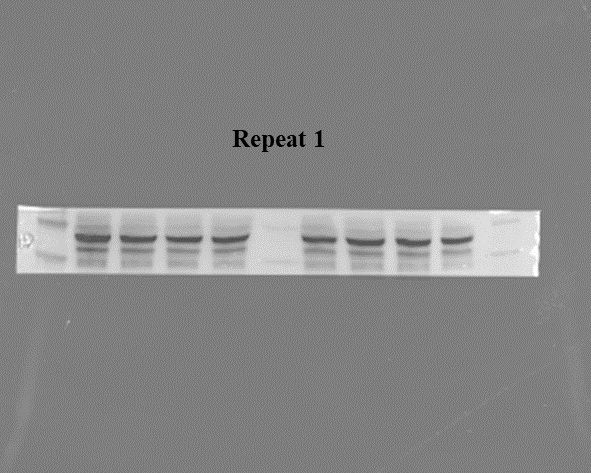


**Figure 4 Full-length blots and original blots generated alternative repeats of western bloting of Figure 5C.**

Whole membrane：

CD206: 245KD

CD68:100KD

CD86：70KD

β-Actin: 42KD

**Figure 5 Full-length blots and original blots generated alternative repeats of western bloting of Figure 5I.**

Whole membrane：

iNOS：130KD

Cleaved caspase3: 17,19KD

β-Actin: 42KD

**Figure 6 Full-length blots and original blots generated alternative repeats of western bloting of Figure 6B.**

Whole membrane：

iNOS：130KD

Arg1:40KD

β-Actin: 42KD

**Figure 7 Full-length blots and original blots generated alternative repeats of western bloting of Figure 7C.**

Whole membrane：

P65：65KD

P-P65: 65KD

IKBα: 39KD

P-IKBα: 39KD

β-Actin: 42KD

**Figure 8 Full-length blots and original blots generated alternative repeats of western bloting of Figure 7D.**

Whole membrane：

JAK1: 130KD

P-JAK1: 130KD

STAT6: 110KD

P-STAT6: 110KD

STAT3: 87KD

P-STAT3: 87KD

β-Actin: 42KD

**Figure 9 Full-length blots and original blots generated alternative repeats of western bloting of Figure 7E.**

Whole membrane：

PI3K: 110KD

P-PI3K: 110KD

AKT: 60KD

P-AKT: 60KD

β-Actin: 42KD

**Figure 10 Full-length blots and original blots generated alternative repeats of western bloting of Figure 8C.**

Whole membrane：

PTEN: 71KD

AKT: 60KD

P-AKT: 60KD

STAT3: 87KD

P-STAT3: 87KD

iNOS：130KD

Arg1: 40KD

β-Actin: 42KD

**Figure 11 Full-length blots and original blots generated alternative repeats of western bloting of Figure 8E.**

Whole membrane：

NLRP3: 110KD

iNOS：130KD

Arg1: 40KD

β-Actin: 42KD

**Figure 12 Full-length blots and original blots generated alternative repeats of western bloting of Figure 8G.**

Whole membrane：

mTOR: 289KD

P65：65KD

P-P65：65KD

IKBα: 39KD

P-IKBα: 39KD

iNOS：130KD

Arg1: 40KD

β-Actin: 42KD

**Figure 13 Full-length blots and original blots generated alternative repeats of western bloting of Figure 8I.**

Whole membrane：

TLR3: 130KD

STAT3: 87KD

P-STAT3: 87KD

P65：65KD

P-P65：65KD

IKBα: 39KD

P-IKBα: 39KD

iNOS：130KD

Arg1: 40KD

β-Actin: 42KD

**Figure 14 Full-length blots and original blots generated alternative repeats of western bloting of Figure 8L.**

Whole membrane：

PTEN: 71KD

AKT: 60KD

P-AKT: 60KD

STAT3: 87KD

P-STAT3: 87KD

iNOS：130KD

Arg1: 40KD

β-Actin: 42KD
